# Organ-specific proteomic aging clocks predict disease and longevity across diverse populations

**DOI:** 10.1038/s43587-025-01016-8

**Published:** 2025-11-26

**Authors:** Yunhe Wang, Sihao Xiao, Bowen Liu, Rongtao Jiang, Yuxi Liu, Yian Hang, Li Chen, Runsen Chen, Michael V. Vitiello, Derrick Bennett, Baihan Wang, Jun Lv, Canqing Yu, Danielle E. Haslam, Qianyan Zheng, Robert E. Gerszten, Yanping Bao, Jie Shi, Junqing Xie, Lin Lu, Liming Li, Cornelia M. van Duijn, Dong D. Wang, Zhengming Chen, Andrew T. Chan

**Affiliations:** 1 Nuffield Department of Population Health, University of Oxford, Oxford, UK; 2 Broad Institute of MIT and Harvard, Cambridge, MA USA; 3 Channing Division of Network Medicine, Department of Medicine, Brigham and Women’s Hospital and Harvard Medical School, Boston, MA USA; 4 State Key Laboratory of Cognitive Neuroscience and Learning, Beijing Normal University, Beijing, China; 5 Department of Radiology and Biomedical Imaging, Yale School of Medicine, New Haven, CT USA; 6 Department of Epidemiology, Harvard T.H. Chan School of Public Health, Boston, MA USA; 7 Department of Biology, University of Oxford, Oxford, UK; 8 School of Public Health, Peking University, Beijing, China; 9 Vanke School of Public Health and Institute for Healthy China, Tsinghua University, Beijing, China; 10 Department of Psychiatry and Behavioral Sciences, University of Washington School of Medicine, Seattle, WA USA; 11 Peking University Center for Public Health and Epidemic Preparedness and Response, Beijing, China; 12 Key Laboratory of Epidemiology of Major Diseases (Peking University), Ministry of Education, Beijing, China; 13 Department of Nutrition, Harvard T.H. Chan School of Public Health, Boston, MA USA; 14 Division of Cardiovascular Medicine, Beth Israel Deaconess Medical Center, Boston, MA USA; 15 National Institute on Drug Dependence, Peking University, Beijing, China; 16 Centre for Statistics in Medicine and NIHR Biomedical Research Centre Oxford, NDORMS, University of Oxford, Oxford, UK; 17 Institute of Mental Health, National Clinical Research Center for Mental Disorders, Key Laboratory of Mental Health and Peking University Sixth Hospital, Peking University, Beijing, China; 18 Peking-Tsinghua Center for Life Sciences and PKU-IDG/McGovern Institute for Brain Research, Peking University, Beijing, China; 19 Clinical and Translational Epidemiology Unit, Massachusetts General Hospital and Harvard Medical School, Boston, MA USA

**Keywords:** Neurological disorders, Psychiatric disorders, Endocrine system and metabolic diseases, Predictive markers, Ageing

## Abstract

Aging and age-related diseases share convergent pathways at the proteome level. Here, using plasma proteomics and machine learning, we developed organismal and ten organ-specific aging clocks in the UK Biobank (*n* = 43,616) and validated their high accuracy in cohorts from China (*n* = 3,977) and the USA (*n* = 800; cross-cohort *r* = 0.98 and 0.93). Accelerated organ aging predicted disease onset, progression and mortality beyond clinical and genetic risk factors, with brain aging being most strongly linked to mortality. Organ aging reflected both genetic and environmental determinants: brain aging was associated with lifestyle, the *GABBR1* and *ECM1* genes, and brain structure. Distinct organ-specific pathogenic pathways were identified, with the brain and artery clocks linking synaptic loss, vascular dysfunction and glial activation to cognitive decline and dementia. The brain aging clock further stratified Alzheimer’s disease risk across *APOE* haplotypes, and a super-youthful brain appears to confer resilience to *APOE4*. Together, proteomic organ aging clocks provide a biologically interpretable framework for tracking aging and disease risk across diverse populations.

## Abstract (web summary)

Wang, Xiao and colleagues develop and validate organ-specific proteomic aging clocks across large population cohorts in the UK, the USA and China, which show strong performance in tracking organ aging and predicting the risk of morbidity and mortality.

## Main

Aging is a continuous process of functional loss that increases susceptibility to various diseases and ultimately leads to death^1^. This complex biological process, shaped by both environmental and genetic factors, shows substantial heterogeneity—not only among individuals of the same chronological age but also across different cells, tissues and organs within a single individual^2–4^. Accurate, systematic measurement of biological aging is, therefore, essential for tracking the aging process, understanding age-related diseases, and evaluating responses to lifestyle and pharmacological interventions^5^. Substantial progress has been made in assessing overall organismal aging through the development of aging clocks based on diverse clinical or omics-based biomarkers^4,6,7^. However, these studies have often lacked detailed characterization of aging dynamics across multiple organs or physiological subsystems^4,6,7^. While several organ-specific clocks have been developed based on imaging phenotypes and clinical biomarkers of organ functions^4,8,9^, many of these clocks demonstrate limited organ specificity, largely reflecting systemic or overall aging rather than capturing organ-intrinsic aging processes. Moreover, although these clocks have been associated with age-related phenotypes^8,9^, they often fall short in providing molecular insights or establishing mechanistic links to the known pathways underlying aging and age-related diseases.

Loss of proteostasis is a fundamental hallmark of aging and is implicated in numerous age-related conditions, including neurodegenerative and musculoskeletal disorders^1^. As an intermediate layer linking the genome to biological processes and phenotypes, the plasma proteome is more proximal to the downstream mechanisms driving aging than other omics layers, such as DNA methylation (DNAm), which is commonly used in the development of aging clocks^6^. Measuring thousands of circulating proteins, plasma proteomics offers a powerful approach to gain molecular-level insights into the aging process and related biology^6^. A recent study has demonstrated that organ-enriched proteins derived from aptamer-based plasma proteomics can quantify biologically interpretable organ-specific aging and predict disease risk^3^. However, several critical questions remain. The reproducibility of organ-specific aging measures across broader populations and the applicability of antibody-based proteomic platforms^10^ for this purpose have yet to be established. Moreover, the extent to which a single organ-specific aging clock provides predictive value independent of other organ clocks and established clinical and genetic biomarkers has not been fully evaluated. Notably, many existing aging clocks targeting specific systems, such as the brain or immune system, rely on hundreds of proteins^3^, limiting their feasibility for clinical translation. A parsimonious panel comprising a minimal number of proteins while retaining strong predictive performance—akin to how individual plasma protein biomarkers (for example, B-type natriuretic peptide for cardiac function and alanine aminotransferase for liver damage) are used in clinical practice—would substantially enhance translational potential.

To address these knowledge gaps, we leveraged the largest proteomic dataset to date from the UK Biobank (UKB; *n* = 43,616) to construct proteomic aging clocks at both the organismal and organ-specific levels across ten major organ systems, using nonlinear machine learning methods. We externally validated these models in two cohorts with distinct ethnic and geographic backgrounds: the China Kadoorie Biobank (CKB; *n* = 3,977) and the US-based Nurses’ Health Study (NHS; *n* = 800). We comprehensively profiled the contributions of environmental, lifestyle and genetic factors to organ aging and examined the associations between organ aging and brain structural features. We also systematically assessed the prospective relationships between organ-specific aging and age-related phenotypes, chronic diseases and all-cause mortality across the three cohorts. Furthermore, we prioritized key organs and proteins implicated in aging and disease risk, particularly neurodegenerative conditions—highlighting shared molecular pathways that may underlie both organ aging and disease onset. Finally, we developed parsimonious models using a reduced set of proteins that retained predictive performance comparable to that of the full models, enhancing their potential for clinical application. Focusing on neurodegenerative diseases (NDs), our findings demonstrate that proteomic organ aging clocks offer a noninvasive and interpretable tool for quantifying organ aging and predicting disease and mortality risk across diverse populations.

## Results

### Proteomic clocks capture organ-specific aging and ageotypes across diverse populations

**Extended Data Fig. 1:**
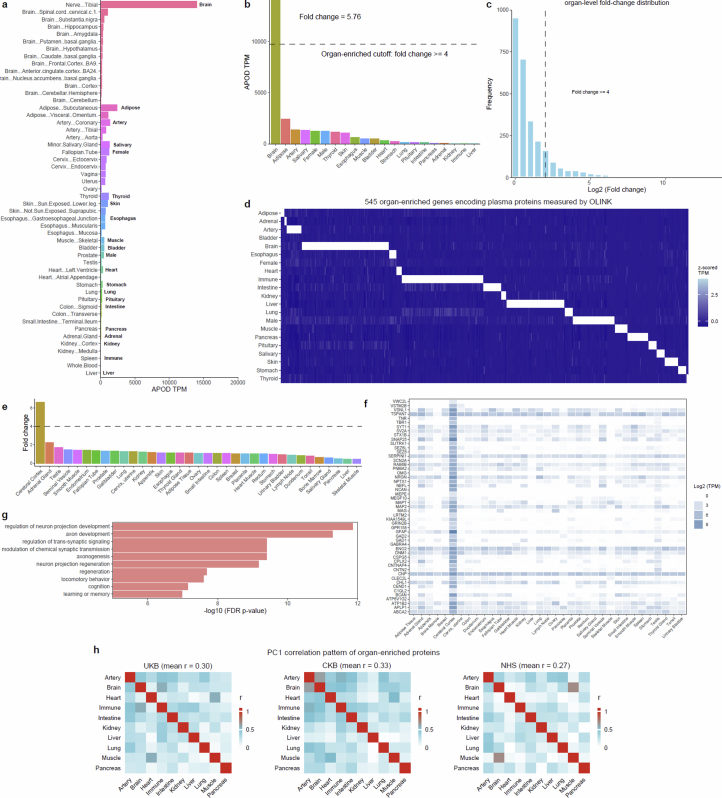
Identification of organ-enriched genes and plasma proteins. **a**, Tissue to organ mapping in GTEx. Tissues were mapped to corresponding organs according to the physiological function. Organ-level gene expression was established by identifying the maximum expression value among its tissue subtypes. Expression levels of APOD across tissues were illustrated as an example. **b**, Organ-level expression of APOD in GTEx. APOD shows a ≥ 4-fold higher expression in the brain than in other organs and is defined as brain-enriched. **c**, Organ-level fold-change distribution of Olink protein encoding genes in GTEx. **d**, Organ-level expression of 545 organ-enriched genes encoding Olink proteins in GTEx. Organ-enriched genes are denoted in white. **e**, Tissue-level expression of identified brain-enriched genes in HPA. **f**, Tissue-level expression of identified individual brain-enriched genes in HPA. **g**, Top 10 most FDR significant enrichment biological pathway enrichment for the identified brain-enriched proteins. Functional enrichment analysis was performed using GO terms and enriched GO terms were identified using a hypergeometric test and corrected for multiple testing. **h**, PC1 correlation pattern of organ-enriched proteins across cohorts. Overlap in numbers of protein signatures between the Olink-based organ aging clock and the SOMAscan-based clock by Oh et al.^3^ was shown in Supplementary Fig. 5. Source Data

Our study included 43,616 participants from the UKB (54% women, baseline age range: 37–70 years) and two independent external validation cohorts: 3,977 Chinese participants from the CKB (54% women, aged 30–78 years) and 800 US participants from the NHS (100% women, aged 43–69 years) (Supplementary Table 1). Plasma proteomic profiling was conducted in all three cohorts using the Olink Explore 3072 panel, measuring 2,916 proteins (Methods). To identify organ-specific proteins, we integrated tissue-level expression data from the Genotype–Tissue Expression (GTEx) project^11^ and annotated 418 proteins (14.3%) as enriched in at least one of ten major organs or systems, including the brain, heart, lung, immune system, artery, intestine, liver, kidney, muscle and pancreas (Extended Data Fig. 1 and Supplementary Tables 2–4). The brain and immune system had the highest number of enriched proteins (117 and 109, respectively). We quantified the overall configuration of organ-specific protein profiles using the first principal component (PC1) for each organ. Across different organs, these PC1s showed low to moderate correlations, suggesting partial independence in organ-specific proteins (Extended Data Fig. 1 and Supplementary Fig. 1).

**Fig. 1:**
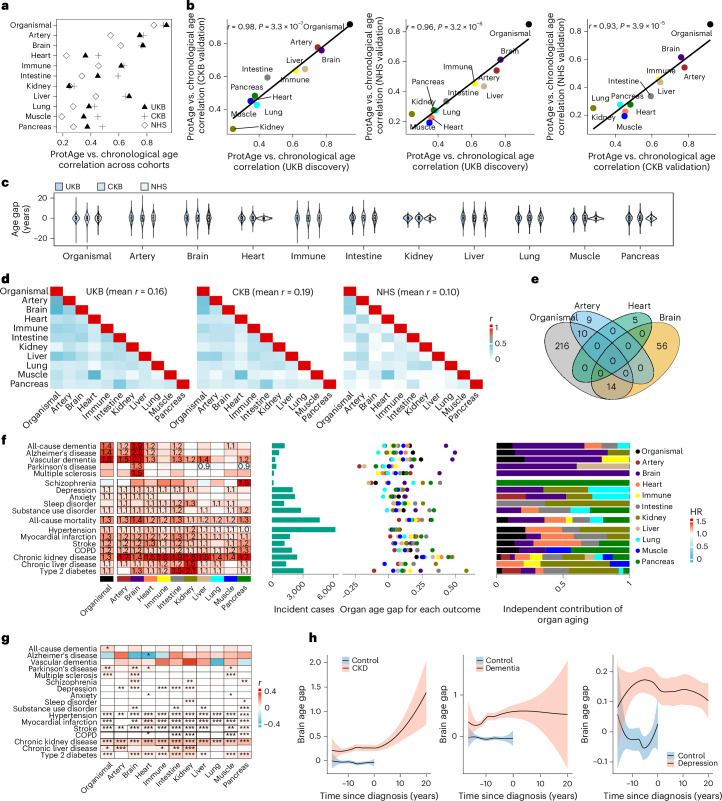
Organ-specific proteomic aging clocks and their associations with disease and mortality across diverse populations. **a**, Performance of the organ aging models across the discovery cohort (UKB, *n* = 43,616) and external validation cohorts (CKB, *n* = 3,977; NHS, *n* = 800). Models were trained on organ-enriched proteins from the Olink Explore 3072 panel, which were identified by GTEx tissue expression data. Performance was assessed using Pearson correlations between predicted organ age and chronological age. The top 20 proteins included in each model are detailed in Extended Data Fig. 2. **b**, Cross-cohort consistency of the performance of proteomic organ aging clocks, assessed using Pearson correlation (left, UKB versus CKB; middle, UKB versus NHS; right, CKB versus NHS). **c**, Distribution of proteomic organ age gap across cohorts. Box bounds indicate the first quartile (Q1), median and Q3; whiskers extend to Q1 − 1.5 × interquartile range (IQR) and Q3 + 1.5 × IQR. **d**, Pairwise correlations among organismal and organ-specific age gaps in the UKB (mean *r* = 0.16), CKB (mean *r* = 0.19) and NHS (mean *r* = 0.10). **e**, Overlap in constituent proteins among the organismal aging clock and three representative organ-specific clocks (brain, artery and heart). **f**, Associations between organ-specific age gaps and the incidence of five NDs, five psychiatric disorders, seven other chronic physical diseases and all-cause mortality in the UKB (*n* = 43,616). Associations were externally validated in the CKB and NHS (Extended Data Fig. 3). HRs per 1-s.d. change in the organismal and ten organ-specific age gaps are shown for significant associations, estimated using separate Cox proportional hazards models for each outcome, with adjustments for age, sex, ethnicity, Townsend deprivation index, smoking, physical activity level and recruitment center. The number of incident cases is presented. Mean differences in organ age gaps at baseline between participants with and without corresponding ‘incident’ diseases are visualized. The right panel shows the relative contributions of organ age gaps to each outcome, calculated by scaling *z*-scores for significant organs so that they sum to 1. **g**, Association between organ age gaps and years since disease diagnosis in participants with prevalent diseases at the baseline proteomic assessment, assessed by Pearson correlation. **h**, Visualization of the brain age gap after prevalent diseases before baseline (reflecting disease progression) or before incident diseases (reflecting prodromal disease). Participants with incident diseases were matched by age (±2 years) and sex with five healthy controls without corresponding incident diseases during the follow-up. The associations of brain age gaps with CKD (*n* = 11,890), ACD (*n* = 5,760) and depression (*n* = 9,710) are shown as examples. Trajectories were fitted using Loess regression, with error bands indicating 95% confidence intervals (CIs). All regression models were adjusted for age, sex, ethnicity, Townsend deprivation index, smoking, physical activity level and recruitment center. All statistical tests are two-sided. The Benjamini–Hochberg FDR was used to correct for multiple comparisons in **f** and **g**. The asterisks denote FDR-adjusted *P*-value thresholds: *q < 0.05; **q < 0.01; ***q < 0.001. ProtAge, proteomic age; COPD, chronic obstructive pulmonary disease.

**Extended Data Fig. 2:**
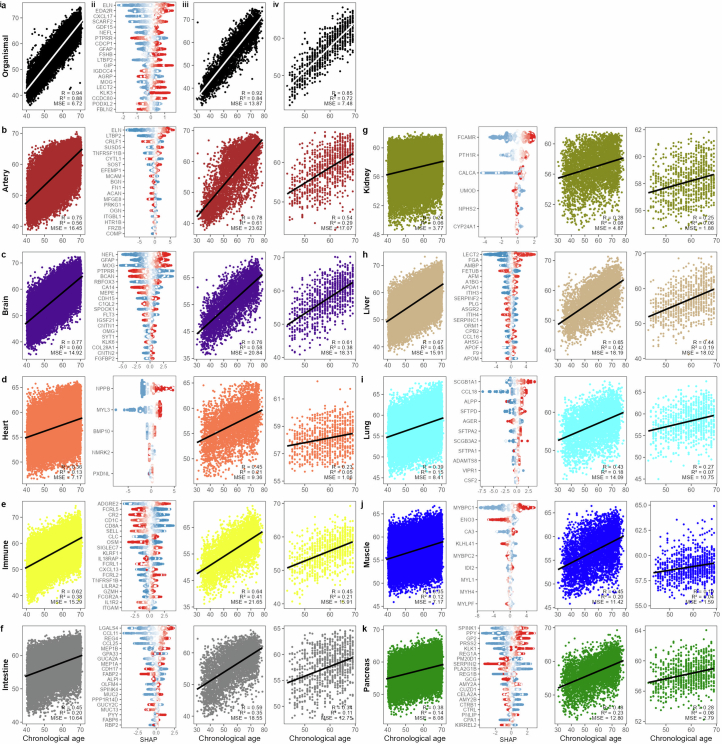
Performance of organ aging models across cohorts and SHAP visualization of selected proteins. **a**-**k**, Organ aging model performance in the discovery UKB cohort (i), the SHAP values of the top 20 selected proteins (ii), model performance in the external validation CKB cohort (iii), and model performance in the external validation NHS cohort (iv). The width of SHAP plot indicates the contribution of proteins to age and color gradient from blue (low) to red (high) indicates the direction of the protein effect on age. The positive direction on x axis indicates proteins are associated with older age, with the negative direction indicating younger age.

For model development, we randomly split the UKB dataset into training and test sets (70:30 ratio). We used the light gradient boosting machine (LightGBM) model with the Boruta feature selection algorithm to identify protein subsets that are predictive of chronological age (Methods). In the training set (*n* = 30,536), we trained one organismal aging model using all proteins and ten organ-specific aging models using the corresponding organ-enriched proteins. The final organismal aging clock included 240 age-related proteins (APs), while the number of APs in the organ-specific aging clocks ranged from 5 (heart) to 76 (immune system). In the UKB test set, protein-predicted organismal age strongly correlated with chronological age (Pearson *r* = 0.94), and organ-specific ages showed moderate to strong correlations (for example, *r* = 0.78 for the brain; Extended Data Fig. 2). Similar patterns were consistently observed in the CKB and NHS, confirming the high age-prediction accuracy of the developed models (Fig. 1b,c and Supplementary Table 5). Based on the proteomic age, we defined two aging phenotypes: (1) age gap (the residual of proteomic age regressed on chronological age), indicating accelerated or delayed aging relative to same-aged peers, and (2) extreme ageotypes, which are identified in individuals with at least one organ age gap beyond ±1.5 s.d., reflecting extremely aged or youthful organs.

To assess heterogeneity in aging across organs, we calculated pairwise correlations of organ age gaps across cohorts (Fig. 1d and Supplementary Table 6). The correlations were generally weak, suggesting a degree of organ specificity in biological aging, with brain and arterial aging showing the strongest correlations with organismal aging despite minimal protein overlap (6% and 4%; Fig. 1e). These findings highlight the potential central roles of brain and vascular aging in systemic aging processes^12,13^.

Collectively, these results indicate that the plasma proteome-based clocks we developed and validated have the potential to robustly capture biological aging at both systemic and organ-specific levels across diverse populations.

### Organ aging clocks predict multiorgan diseases, multimorbidity and mortality

Next, we investigated whether, and to what extent, the associations between organ aging clocks and diseases and mortality differ across organs.

#### Organ age gap, diseases and mortality

In the UKB, the organ age gap was strongly associated with the risk of incident diseases affecting the corresponding organ system (121/187, 65% significant after false discovery rate (FDR) correction; Fig. 1f and Supplementary Table 7), after adjusting for chronological age, sex, and major sociodemographic and lifestyle risk factors. For example, the brain age gap showed the strongest association with the future risk of NDs, including all-cause dementia (ACD; hazard ratio (HR) per 1-s.d. change: 1.88, *q* = 8.15 × 10^−47^), multiple sclerosis (MS; HR per 1-s.d. change: 1.52, *q* = 2.21 × 10^−4^) and Parkinson’s disease (PD; HR per 1-s.d. change: 1.30, *q* = 5.48 × 10^−4^). The kidney and intestine age gaps were most significantly associated with the risk of incident chronic kidney disease (CKD; HR per 1-s.d. change: 1.78, *q* = 3.31 × 10^−78^ for kidney; HR per 1-s.d. change: 1.93, *q* = 1.45 × 10^−98^ for intestine) and type 2 diabetes (T2D; HR per 1-s.d. change: 2.08, *q* = 5.93 × 10^−152^ for kidney; HR per 1-s.d. change: 1.54, *q* = 4.47 × 10^−65^ for intestine). Notably, organ aging also predicted diseases beyond their respective systems; for example, brain aging was linked to multiple physical diseases such as stroke and myocardial infarction. Heart and arterial aging were closely associated with cardiovascular diseases and chronic liver diseases.

Organ-specific aging was more strongly associated with most disease outcomes than organismal aging (Fig. 1f). All organ age gaps predicted all-cause mortality, with a 10–40% higher risk per 1-s.d. increase; the brain showed the strongest effect (HR per 1-s.d. change: 1.44, *q* = 3.35 × 10^−74^). Individuals who later developed Alzheimer’s disease (AD) exhibited the largest baseline brain age gap, whereas the organismal aging was modest (mean: 2.2 versus 0.5 years). Similarly, those with incident CKD had a larger kidney age gap (1.0 versus 0.6 years). These findings indicate that focusing solely on organismal aging^7^ may obscure critical organ-specific contributions to disease risk and mortality, while each organ’s aging trajectory contributes uniquely to overall health.

We then quantified the relative contribution of aging in each organ to the risk of incident diseases (Fig. 1f). NDs were mainly driven by brain aging, which accounted for 45–100% of the associations with organ aging. By contrast, peripheral diseases and mortality reflected multiorgan contributions: although kidney and heart aging showed strong effects for CKD and cardiovascular diseases, respectively, as expected, they explained only part of the associations (for example, kidney aging explained 38% and 17% of the associations with T2D and CKD, respectively). Notably, brain aging was associated with 14 of 17 outcomes independent of other organs, including the strongest association with mortality. These distinct patterns within the multiorgan disease network underscore the pivotal role of brain aging in both NDs and peripheral diseases, as well as its contribution to a healthy lifespan.

**Extended Data Fig. 3:**
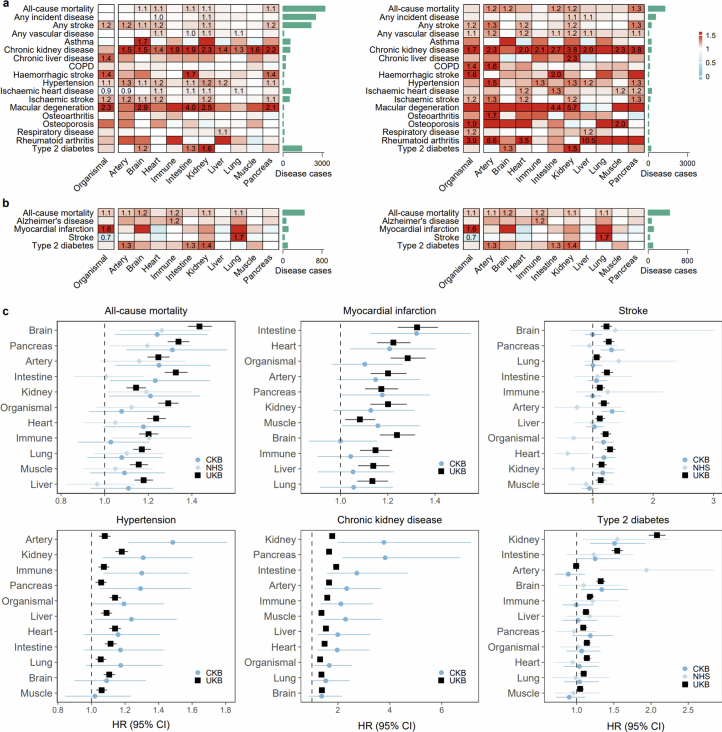
External validation of associations between proteomic organ aging clocks and diseases and mortality in the CKB and NHS. **a**, Association of organ aging clocks with incident diseases and mortality in the full sample of the IHD case-cohort study in the CKB (*n* = 3,977; left panel) and in a subsample excluding enriched IHD patients (*n* = 2,029; right panel). Cox regression models were adjusted for age, sex, ethnicity, education, study region, smoking, and physical activity level. **b**, Association of organ aging clocks with incident diseases and mortality in the full sample of the colon cancer case-control study in the NHS (*n* = 774; left panel) and in a subsample of the control group (*n* = 387; right panel). Cox regression models were adjusted for age, ethnicity, neighborhood socioeconomic status, smoking, and physical activity level. **c**, Comparison of the association between organ aging clocks with incident diseases and death across cohorts. To account for potential bias from the case-control design, UKB (*n* = 43,616) estimates were compared with those from the control groups of the CKB (*n* = 2,029) and NHS (*n* = 387). Squares represent HRs, and error bars represent the corresponding 95% CIs. Detailed results are presented in Supplementary Tables 8, 9.

Importantly, the observed associations in the UKB were replicated in two independent cohorts (CKB, with 11–16 years of follow-up; NHS, with 30 years of follow-up). These included the robust associations of brain aging with all-cause mortality, kidney aging with CKD and T2D, as well as artery, heart and kidney aging with myocardial infarction and hypertension (Extended Data Fig. 3 and Supplementary Tables 8 and 9).

#### Disease progression and organ age gap

Given that prevalent chronic diseases can accelerate biological aging, we examined the organ age gap in relation to disease progression, defined as the time since diagnosis in participants with prevalent diseases at baseline (Fig. 1g and Supplementary Table 10). Most physical diseases (for example, CKD, T2D and stroke) were linked to accelerated aging across nearly all organs. In contrast, NDs had minimal effects, except for PD and MS, which were associated with organismal, brain and muscle aging, consistent with their hallmark motor symptoms. We further assessed the brain age gap during the prodromal phase (from baseline to disease onset) by comparing incident cases with matched healthy controls. We found that brain aging progressed with CKD and depression but not with dementia, despite signs of accelerated aging before the onset of these diseases (Fig. 1h). These findings indicate that organ aging clocks capture changes in biological aging both before and after the onset of physical diseases; however, the brain clock specifically captures changes that precede—but likely not those that follow—the onset of dementia. After excluding all baseline diseases that accelerate organ aging, the organ age gap still strongly predicted diseases and death (Supplementary Fig. 2), suggesting its robustness against potential reverse causality from prevalent diseases.

#### Organ ageotypes, incident diseases and mortality

**Extended Data Fig. 4:**
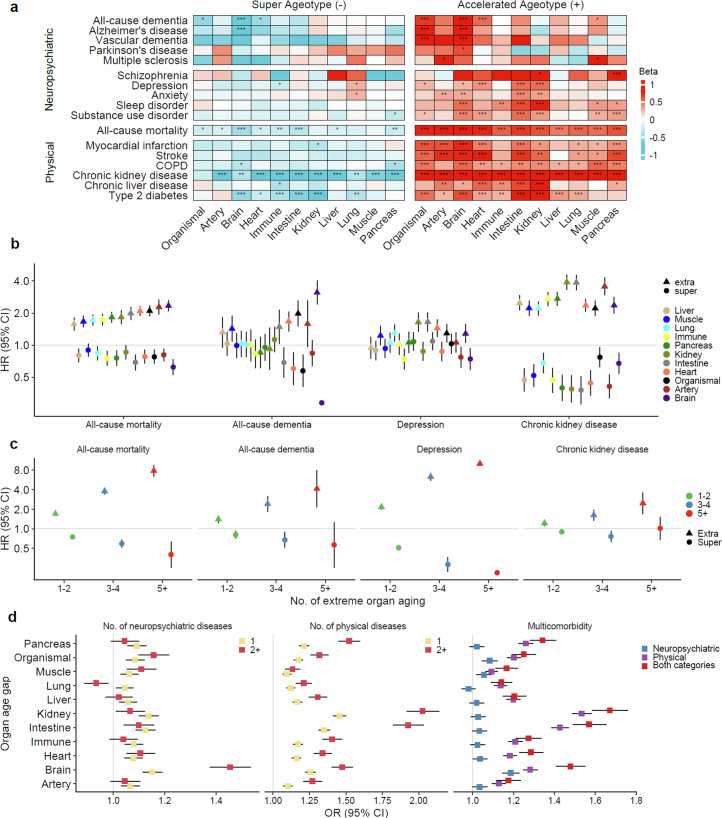
Association of extreme ageotypes across multiple organs with diseases, multimorbidity, and mortality. **a**, Overall association of extreme ageotypes (super ager and accelerated ager) with diseases and death (*n* = 43,616), assessed by Cox models. Accelerated ager and super ager was defined as ± 1.5 standard deviations beside the mean of z-scored age gap for at least one organ, respectively. In the UKB, 25% and 15% of participants had one or multiple extremely aged organs, respectively, while 27% and 20% had one or multiple extremely youthful organs. 14% had both extremely aged and youthful organs. **b**, Visualization of the organ-specific extreme ageotypes in relation to all-cause mortality, all-cause dementia, depression, and CKD (*n* = 43,616). Organ-specific models were ordered by the association of accelerated ageotypes with mortality for each outcome. **c**, Association of number of extreme ageotypes (super/extra) with mortality, dementia, depression, and chronic kidney disease. **d**, Association of organ age gap with multimorbidity. Multimorbidity is defined as having two or more incident neuropsychiatric diseases (left panel; 3,982 and 1,621 participants with one or two incident neuropsychiatric diseases), two or more other incident chronic diseases studied (middle panel; 6,658 and 2,116 participants with one or two incident physical diseases), or both incident neuropsychiatric and chronic diseases (right panel; 2,044 multimorbidity cases) during follow-up. Squares represent effect sizes (ORs, or HRs) and error bars the corresponding 95% CIs in **b**-**d**. All regression models were adjusted for age, sex, ethnicity, Townsend deprivation index, smoking, physical activity level, and recruitment center. The asterisks denote FDR-adjusted P value thresholds: *q < 0.05; **q < 0.01; ***q < 0.001. Error bars indicate 95% CIs.

We then evaluated the association of extreme organ ageotypes with diseases and mortality in the UKB (Extended Data Fig. 4a,b and Supplementary Table 11), finding trends largely consistent with those observed for organ age gaps. Ageotypes defined by accelerated organ aging were associated with an increased risk of most physical diseases, mortality and several neuropsychiatric disorders, with accelerated brain aging linked to most outcomes (15 of 17). Super-youthful ageotypes showed fewer significant associations with outcomes than accelerated ageotypes. The number of extreme organs showed a dose-dependent relationship with the risk of diseases and death (Extended Data Fig. 4c). Compared to participants without any extreme organs, those with one to two, three to four, and five or more extremely aged organs had a 1.7-, 3.8- and 7.8-fold higher mortality risk, while those with the same number of extremely youthful organs had a 25%, 40% and 60% lower risk, respectively. Dementia risk similarly increased with more aged organs (1.4-, 2.4- and 4.1-fold) and decreased with more youthful organs (by 19%, 49% and 75%).

#### Organ age gap and incident multimorbidity

We examined the association between organ age gap and multimorbidity, defined as two or more incident diseases within the neuropsychiatric and/or chronic physical categories (Extended Data Fig. 4d). For neuropsychiatric disorders, the associations with single diseases and multimorbidity were similar across organs, except for the brain. Brain aging was more strongly linked to neuropsychiatric multimorbidity (odds ratio (OR) = 1.45) than to a single disease (OR = 1.15). For physical diseases, aging across all organs was more strongly associated with multimorbidity than with single diseases. These findings suggest that brain aging may uniquely underlie neuropsychiatric multimorbidity, while chronic physical diseases and their comorbidities are more broadly driven by aging across multiple organ systems. Organ aging was also strongly linked to incident multimorbidity of both physical and neuropsychiatric diseases, with the kidney, intestine, pancreas and brain being the top organs involved. This highlights the central role of aging in the brain and key digestive and endocrine organs in connecting brain and body diseases, consistent with the brain–gut connection in mental and physical health^14,15^. These results align with recent findings suggesting that aging in one specific organ can increase susceptibility to comorbidities across multiple organs^16^.

### Genetic and environmental links to organ aging

#### Clinical biomarkers, age-related traits and organ aging

**Fig. 2:**
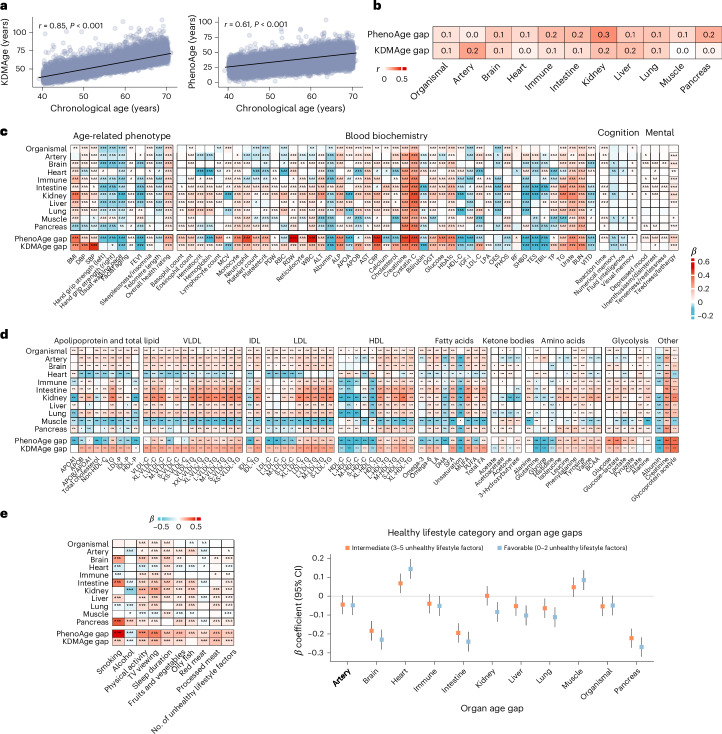
Association of proteomic organ aging clocks (versus established phenotypic aging clocks) with age-related traits, clinical markers, metabolites and lifestyle factors. **a**, Pearson correlation of phenotypic age (KDMAge and PhenoAge) with chronological age. KDMAge and PhenoAge were trained using data from the National Health and Nutrition Examination Survey with an established algorithm and then mapped to the UKB data. **b**, Proteomic organ age gaps were only weakly correlated with established phenotypic age gaps. **c**, Association of proteomic organ age gaps and phenotypic age gaps with age-related traits, clinical markers, and cognitive and mental health measures. **d**, Associations of proteomic organ aging clocks with plasma metabolites measured using an NMR-based metabolomics platform. The clocks are broadly associated with an atherogenic metabolite profile. **e**, Associations of proteomic organ aging clocks with modifiable lifestyle factors (smoking, alcohol consumption, physical activity, TV watching/sedentary time, sleep duration, and intake of fruits and vegetables, oily fish, red meat and processed meat; *n* = 43,616). Associations with individual lifestyle factors (left) and with lifestyle risk categories based on the number of unhealthy factors (favorable: 0–2, intermediate: 3–5, unfavorable: 6–9) (right) are shown. Squares represent *β* coefficients, and error bars indicate the corresponding 95% CIs. Panels **c**–**e** display *β* coefficients from linear regression models (adjusted for age, sex, ethnicity, Townsend deprivation index, smoking, physical activity level and recruitment center in **c** and **d**; adjusted for age, sex, ethnicity, Townsend deprivation index and recruitment center in **e**). All statistical tests are two-sided. The Benjamini–Hochberg FDR was used to correct for multiple comparisons in **c**–**e**. The asterisks denote FDR-adjusted *P*-value thresholds: *q < 0.05; **q < 0.01; ***q < 0.001. Abbreviations are defined in Supplementary Table 13.

We investigated the association of organ age gaps with 11 physiological phenotypes, 8 cognitive/mental health measures and 61 blood chemistry markers. After adjusting for confounders, both organ and organismal aging were linked to various age-related physiological phenotypes, including higher body mass index and blood pressure, a greater likelihood of sleeplessness, shorter telomeres, slower walking speed, and poorer cognitive, mental and overall health (Fig. 2c). We also observed associations between organ aging and multiple blood biomarkers, including elevated levels of blood urea nitrogen (indicating kidney dysfunction), albumin (an indicator of liver or kidney disease), alanine aminotransferase to aspartate aminotransferase ratio (a marker of liver damage), creatinine (indicating kidney injury) and C-reactive protein (a marker of inflammation). Additionally, organ aging was linked to atherosclerotic lipid profiles and higher glucose levels (Fig. 2d). Lifestyle factors, including behavioral and dietary habits, were strongly associated with organ aging in a cross-sectional analysis (Fig. 2e). Unhealthy behaviors were associated with accelerated aging, especially in the brain and pancreas, while greater adherence to a healthy lifestyle was linked to slower aging in organs such as the brain and intestine. See Supplementary Note 1 for full details.

#### Genetic determinants of organ aging clocks

**Extended Data Fig. 5:**
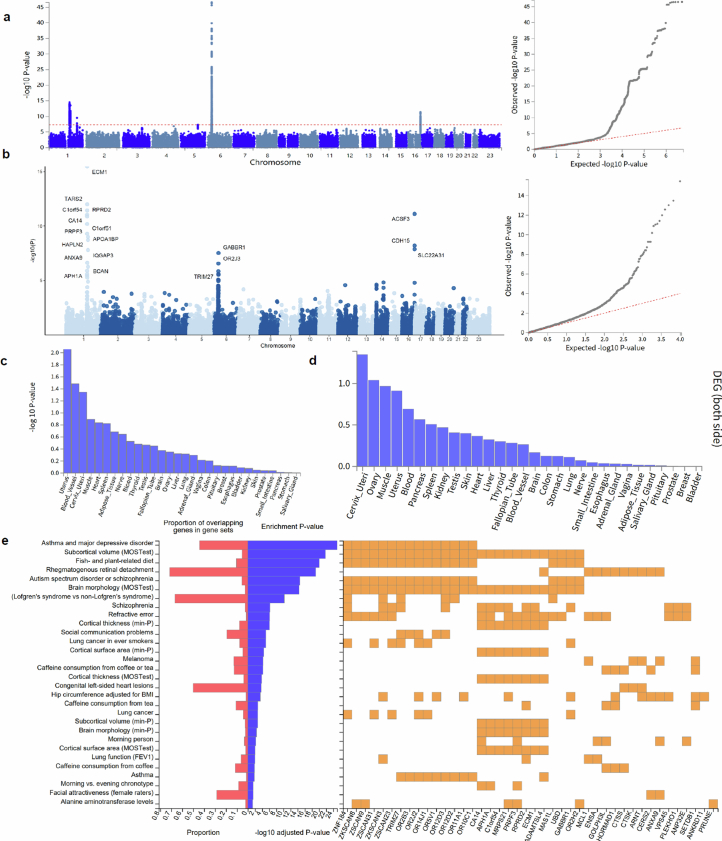
Genetic determinants of brain aging clock with functional mapping and annotation. **a**, Manhattan and QQ plots of GWAS on brain aging clock. Independent genome-wide significant SNPs were shown in Supplementary Table 15. **b**, Manhattan and QQ plots of MAGMA gene-based analysis from GWAS on brain aging clock. Input SNPs were mapped to 19839 protein coding genes. Significant genes are shown (*P* < 0.05/19839). **c**, MAGMA tissue enrichment analysis of brain ageing clock-related genes in 30 general tissue types from GTEx v8. **d**, Enrichment test of prioritized genes in differentially expressed gene (DEG) sets from GTEx v8 30 general tissue types. DEG at both sides are shown. No significant tissue enrichment was observed. **e**, Overlap and enrichment of input genes in GWAS catalog reported gene set (Supplementary Table 16).

**Extended Data Fig. 6:**
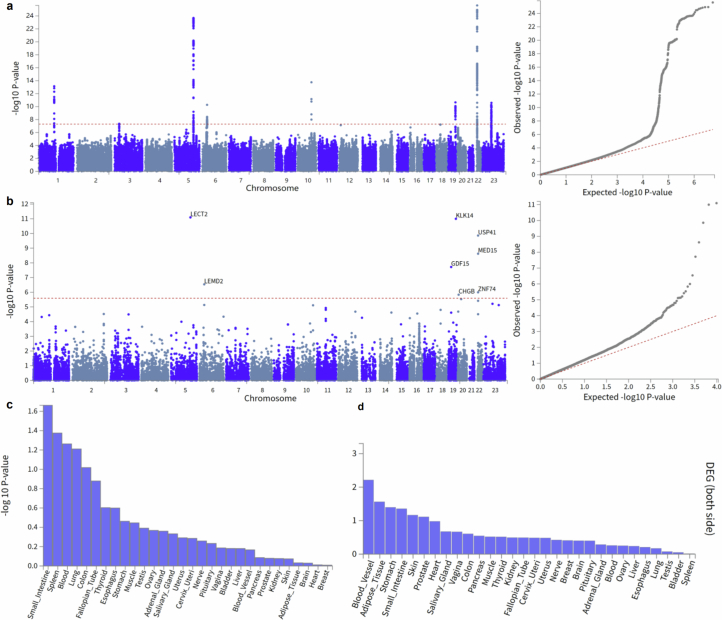
Genetic determinants of organismal aging clock with functional mapping and annotation. **a**, Manhattan and QQ plots of GWAS on organismal aging clock. Independent genome-wide significant SNPs were shown in Supplementary Table 17. **b**, Manhattan and QQ plots of MAGMA gene-based analysis from GWAS on organismal aging clock. Input SNPs were mapped to 19839 protein coding genes. Significant genes are shown (*P* < 0.05/19839). **c**, MAGMA tissue enrichment analysis of organismal ageing clock-related genes in 30 general tissue types from GTEx v8. No significant tissue enrichment was observed. **d**, Enrichment test of prioritized genes in differentially expressed gene (DEG) sets from GTEx v8 30 general tissue types. DEG at both sides are shown. Overlap and enrichment of input genes in GWAS catalog reported gene sets were shown in Supplementary Table 18.

We conducted genome-wide association studies (GWASs) on brain and organismal age gaps in 29,629 UKB participants of European ancestry. For the brain age gap, we identified 38 independent genome-wide significant single nucleotide polymorphisms (SNPs) at six genomic risk loci, which were mapped to 149 protein-coding genes (Extended Data Fig. 5). The top-ranked genes included *GABBR1* (refs. ^17–20^), *ECM1* (ref. ^21^), *TARS2* (ref. ^22^), *ARNT* (ref. ^23^) and *CA14* (ref. ^24^), all of which have previously been implicated in NDs and brain health. For example, *GABBR1* encodes GABA receptors involved in GABAergic neurotransmission, with central roles in AD and psychiatric disorders. Notably, GABA receptors represent potential therapeutic targets for cognitive symptoms and neuropsychiatric disorders^17–20^. Gene set enrichment suggested an overlap with neuropsychiatric disorders and brain morphology. Tissue enrichment analysis showed no significant overexpression, indicating a systemic genetic basis for brain aging, consistent with a recent GWAS on phenotypic brain aging^25^. For the organismal age gap, 33 independent genome-wide significant SNPs were identified within eight genomic risk loci, mapped to 46 protein-coding genes (Extended Data Fig. 6). The top-ranked genes included *KLHL22* (ref. ^26^), *MED15* (refs. ^27,28^), *SCARF2* (ref. ^29^), *ZNF74* (ref. ^30^) and *SMAD5*, which have known roles in longevity and age-related diseases. Similar to brain aging, no significant tissue-specific enrichment was observed for organismal aging, suggesting a broad, systemic genetic basis. See Supplementary Note 2 for full details.

Overall, these findings highlight the influence of both environmental and genetic factors in shaping biological aging, as captured by proteomic aging clocks.

### Brain and body aging predict future cognitive decline and NDs and link distinct pathogenic pathways

Given the strong associations between brain aging and NDs, we further assessed these associations across different disease stages. Additionally, we characterized shared molecular signatures and pathways connecting brain and body aging with NDs in the UKB.

**Fig. 3:**
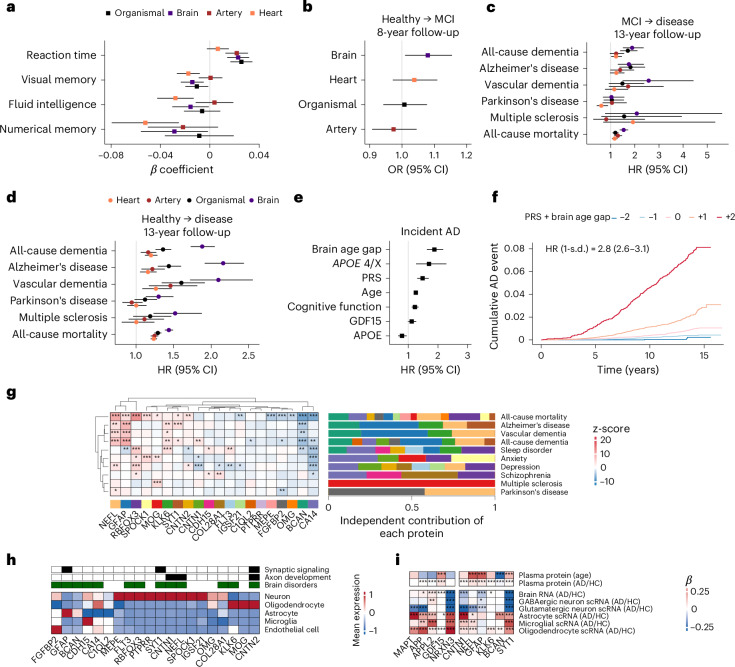
Brain and peripheral organ aging in cognitive decline and NDs. **a**, Associations of brain and peripheral organ (organismal, artery and heart) aging (age gaps) with baseline cognitive function in participants without NDs at baseline (*n* = 43,141). Associations were estimated by linear regression and presented as *β* coefficients. Higher reaction times and lower scores in visual memory, fluid intelligence and numerical memory indicate poorer cognitive function. **b**, Associations of brain and peripheral organ aging with the risk of transitioning from cognitively healthy to MCI (defined as a global cognitive score 1.5 s.d. below the education-adjusted baseline mean in healthy participants) over 8 years of follow-up (*n* = 39,684). Associations were estimated by logistic regression and presented as ORs. **c**, Associations of brain and peripheral organ aging with incident NDs and all-cause mortality in participants with baseline MCI over 13 years of follow-up (*n* = 3,337). **d**, Associations of brain and peripheral organ aging with incident NDs and all-cause mortality in healthy participants over 13 years of follow-up (*n* = 43,616). **e**, Associations between multiple markers (brain age gap, *APOE* ε4 heterozygotes, AD PRS, age, cognitive function, GDF15 and APOE protein) and the risk of incident AD over 13 years of follow-up (*n* = 43,616; 611 events for AD). Associations in **c**–**e** were estimated using Cox models and presented as HRs. Squares/circles represent effect sizes (*β* coefficients, ORs or HRs), and error bars indicate the corresponding 95% CIs in **a**–**e**. **f**, Cumulative incidence curves of AD across combined levels of brain age gap and AD PRS. Participants were grouped into five bins based on the combined standardized scores: bin −2 (<−1.5 s.d.), bin −1 (−1.5 to −0.5 s.d.), bin 0 (−0.5 to +0.5 s.d.), bin +1 (+0.5 to +1.5 s.d.) and bin +2 (>+1.5 s.d.). The displayed HR reflects the AD risk per 1-s.d. increase in the combined scores. **g**, Relative importance of individual proteins in predicting specific disease outcomes. For each disease, Cox models included the top 20 proteins in the brain aging clock, adjusting for covariates. In the left panel, the associations between each protein and incident disease are colored by *z*-score, with *z*-scores for associations with a *P* value of ≥0.05 set to 0. In the right panel, the relative importance of proteins significantly associated with each outcome is displayed. This was calculated as the proportion of each protein’s absolute *z*-scored *β* coefficient relative to the total sum of absolute *z*-scored *β* coefficients for all proteins significantly associated with the given disease. **h**, scRNA expression profiles of the top 20 brain aging proteins in the human brain^40^. Mean normalized expression levels are shown across different cell types. Proteins enriched in the GO pathways GO:0099177 (regulation of trans-synaptic signaling) and GO:0061564 (axon development) are denoted in black. Proteins associated with two or more neuropsychiatric diseases are denoted in green. **i**, Associations of protein levels, bulk RNA expression and scRNA expression with age and AD for key proteins involved in brain aging and dementia risk across tissues (plasma and brain). Associations of plasma protein levels with age or AD were assessed in the UKB (*n* = 43,616) using linear regression (for age) and Cox models (for incident AD). Associations between gene expression in brain tissue and AD were evaluated using logistic regression based on data from ref. ^84^. Results were reported as *β* coefficients. All models were adjusted for age, sex, ethnicity, Townsend deprivation index, smoking, physical activity level and recruitment center in the UKB. All statistical tests are two-sided. The Benjamini–Hochberg FDR was used to correct for multiple comparisons. The asterisks denote FDR-adjusted *P*-value thresholds: *q < 0.05; **q < 0.01; ***q < 0.001. HC, healthy control.

We focused on brain and body (organismal, artery and heart) aging, all of which were independently associated with an increased risk of NDs (Fig. 1f). Both brain and body aging were related to poorer cognitive function across multiple domains in healthy participants at baseline, including reaction time, numerical memory, fluid intelligence and visual memory; brain aging showed significant associations across all cognitive domains assessed (Fig. 3a). Over 8 years of follow-up, the brain age gap was significantly associated with an increased risk of transitioning from cognitively healthy to mild cognitive impairment (MCI) (OR = 1.08, *P* = 0.03) (Fig. 3b). In participants with MCI, both brain and body aging were associated with the risk of subsequent progression to dementia (HR = 1.89, *q* = 3.23 × 10^−8^ for brain aging; HR = 1.71, *q* = 4.46 × 10^−7^ for organismal aging; Fig. 3c), consistent with the associations of incident dementia in healthy individuals at baseline (Fig. 3d). Next, we assessed whether the brain age gap remained predictive of future dementia risk after adjusting for established biomarkers and risk factors, such as chronological age, cognitive function, polygenic risk score (PRS) for AD, *APOE4* genotype and other AD-related proteins not included in the brain aging model, such as GDF15 (ref. ^31^) and APOE^32^ (Fig. 3e). The brain age gap had a strong independent association with dementia (HR = 1.88, *P* = 1.34 × 10^−17^), additive to the effects of other biomarkers such as the *APOE4* genotype (HR = 1.70, *P* = 4.37 × 10^−4^), PRS (HR = 1.48 per 1-s.d. change, *P* = 2.90 × 10^−9^), chronological age (HR = 1.26, *P* = 6.55 × 10^−49^) and cognitive function (HR = 1.23, *P* = 1.73 × 10^−5^). We then assessed the combined effects of brain aging and genetic risk on dementia. The combination of brain age gap and AD PRS stratified the future risk of AD in healthy participants (HR per 1-s.d. change = 2.80, range 2.56–3.06; *P* = 2.00 × 10^−16^). Participants with combined levels of brain age gap and PRS 1 and 2 s.d. above the average were at 2.8 and 9.2 times increased risk of AD, whereas those with combined levels 1 and 2 s.d. below the average were at 55% and 81% lower risk of AD (Fig. 3f).

We then evaluated the relative contributions of individual proteins in the brain aging clock to dementia (Fig. 3g). Among the top 20 proteins in the brain aging clock, those associated with at least two dementia phenotypes included glial fibrillary acidic protein (GFAP), neurofilament light chain polypeptide (NEFL), brevican (BCAN), kallikrein-6 (KLK6) and synaptotagmin-1 (SYT1). GFAP is the most widely used marker for reactive astrocytes^33^, with growing evidence supporting its clinical use in predicting neuroinflammatory disorders and NDs^34^. NEFL is an established marker of neuroaxonal injury, used to monitor disease activity and drug effects in recent clinical trials of neurological diseases^35^. BCAN is integral to the neuroprotective perineuronal nets of the brain extracellular matrix (ECM) that help maintain synaptic functions, and its expression is decreased in patients with vascular dementia (VD)^36^. KLK6 is an age-related protease involved in the proteolysis of extracellular proteins implicated in neurological diseases^37^. SYT1, a presynaptic protein associated with synapse degeneration, has been identified as a biomarker for AD and related cognitive decline^38^, while missense mutations in *SYT1* lead to SYT1-related neurodevelopmental disorders^39^. NEFL, GFAP and BCAN have recently been validated as being associated with future dementia risk in the same UKB cohort^31^. Alterations of proteins in the brain ECM, such as neurocan (NCAN), a member of the lectican family similar to BCAN, were also noted in brain aging^3^.

#### Brain aging in dementia

The top 20 proteins of the brain aging clock were specifically expressed in neurons and glia (oligodendrocytes, astrocytes and microglia), with enrichment in the trans-synaptic signaling regulation and axon development pathways (Fig. 3h). After identifying featured plasma proteins related to both brain aging and dementia, we assessed their expression changes in AD across brain cell types using bulk RNA sequencing (RNA-seq) and single-cell RNA (scRNA)-seq data^40^ (Fig. 3i). We focused on proteins that have been linked to both brain aging and AD in our analysis (NEFL, GFAP, KLK6, BCAN, SYT1 and CNTN1), as well as proteins potentially implicated in AD pathology (MAPT (tau), APP and APPL2) or prioritized in previous studies (GDF15 (ref. ^31^) and NRXN3 (ref. ^3^)). These proteins showed a consistent pattern of reduced expression in the AD brain, particularly in both GABAergic and glutamatergic neurons, while their plasma levels increased with brain aging and AD. In contrast, glial cells showed increased expression of these proteins in patients with AD compared to healthy controls. These patterns are consistent with established AD neuropathology: the decreased levels of proteins related to neurosynaptic growth and glial development (for example, NEFL, SYT1 and GFAP) in neurons and the brain likely reflect neuronal loss and synapse degeneration, accompanied by their subsequent shedding into the bloodstream. A similar inverse pattern between brain and peripheral protein levels has been observed with amyloid β (Aβ), where lower Aβ42 levels in cerebrospinal fluid were associated with a higher brain Aβ burden^3^. The elevated protein expression in glial cells, amid the overall decline in the brain, likely reflects glial activation—a hallmark of AD-related neuroinflammation and disease progression^41,42^. Astrocyte reactivity was also observed, consistent with its proposed role as a critical upstream event linking Aβ accumulation to the initiation of tau pathology in preclinical AD^43^.

Collectively, these findings suggest that the brain aging clock can robustly predict and stratify future dementia risk across different disease stages and *APOE* haplotypes, independently of established biomarkers, while also uncovering distinct pathogenic pathways that link brain aging-related changes to AD.

#### Peripheral organ aging in dementia

**Fig. 4:**
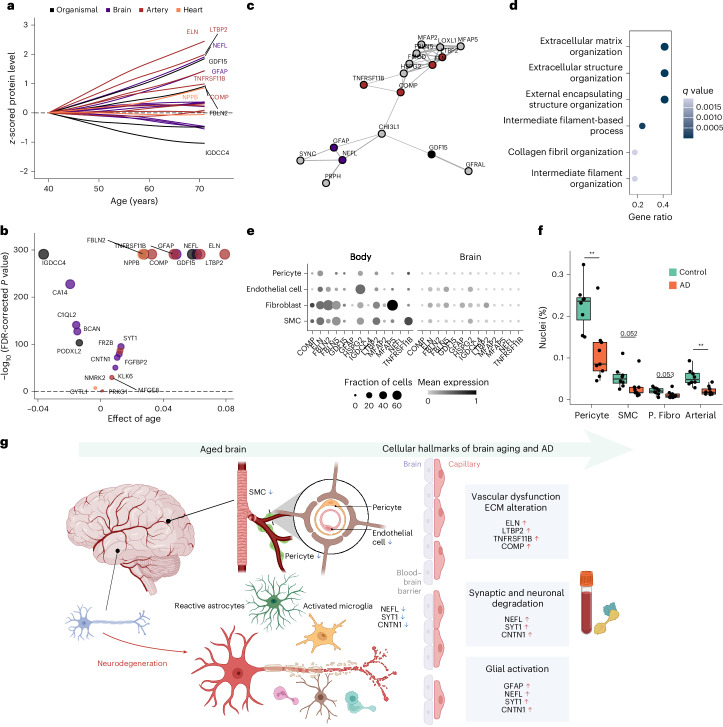
Proteins involved in organismal and organ-specific aging clocks and their associations with age, dementia and vascular biology. **a**, Associations between age and selected key proteins from organismal and organ-specific (brain, artery and heart) aging clocks that were also associated with dementia. The lines were fitted using Loess regression. Proteins in the organismal aging clock are preferentially colored according to their organ specificity when they are also included in organ-specific aging clocks (for example, ELN). Arterial proteins (for example, ELN and LTBP2), organismal proteins (for example, IGDCC4 and GDF15), as well as NEFL and GFAP showed earlier and steeper age-associated increases than other proteins. **b**, Summary of the associations between age and the proteins shown in **a**. Effect estimates from linear regression models (with age as the independent variable and protein levels as the dependent variable) and the corresponding significance levels are shown. **c**, Protein–protein interaction network identified through STRING analysis. Displayed are the interactions of the featured proteins from **a** and **b**, along with their interacting proteins that had a confidence score of ≥0.4. **d**, Enriched biological pathways among proteins involved in aging and dementia. Functional enrichment analysis was performed using GO terms, and enriched GO terms were identified using a hypergeometric test and corrected for multiple testing. **e**, Human scRNA expression of featured proteins in the brain^44^ and peripheral^45^ vasculature. The mean normalized expression and the proportion of cells expressing each gene are shown. These genes are predominantly expressed in endothelial cells, fibroblasts and SMCs in both the brain and peripheral vasculature. **f**, Levels of pericytes, SMCs, perivascular fibroblasts (P. Fibro) and arterial endothelial cells (Arterial) in patients with AD versus healthy controls (*n* = 17). Pericytes (*P* = 0.003), SMCs (*P* = 0.052) and arterial endothelial cells (*P* = 0.002) were decreased in AD, as assessed using the *t*-test. **P < 0.01. Box bounds indicate Q1, median and Q3; whiskers extend to Q1 − 1.5 × IQR and Q3 + 1.5 × IQR. **g**, Schematic model illustrating the contributions of synaptic and neuronal degradation, glial activation, vascular dysfunction and ECM alterations—as captured by the artery and brain aging clocks—to early cognitive impairments and NDs during biological aging. Panel **g** created with BioRender.com.

We then investigated the molecular relevance of body aging beyond the brain in the context of AD. To understand the relative importance of brain and body (artery, heart and organismal) aging in early cognitive decline, we examined the age-associated trajectories of proteins prioritized for their relevance to both aging and dementia (Supplementary Fig. 3a–d). Notably, proteins enriched in arterial aging (for example, ELN and LTBP2) and organismal aging (for example, IGDCC4 and GDF15), as well as NEFL and GFAP, exhibited earlier elevation and steeper age-associated increases than other proteins (Fig. 4a,b). These proteins were highly (and most specifically) expressed in arterial and brain tissues (Supplementary Fig. 3h) and formed a connected protein–protein interaction network in STRING analysis (Fig. 4c), with involvement in ECM and cytoskeletal organization (Fig. 4d). scRNA expression data from the human brain^44^ and peripheral^45^ vasculature revealed that genes encoding these proteins are predominantly expressed by smooth muscle cells (SMCs), fibroblasts and endothelial cells. Loss of these brain vascular cells, such as SMCs, fibroblasts and arterial endothelial cells, has been related to both AD (Fig. 4f)^44^ and the breakdown and dysfunction of the blood–brain barrier, a hallmark of early AD pathophysiology^46^. These findings suggest that early vascular degeneration with aging, including vascular ECM alterations, may reflect both blood–brain barrier disruption and systemic atherosclerotic changes, which are central to the pathogenesis of vascular cognitive impairments^47^, particularly VD. Finally, we propose models in which synaptic and neuronal degradation, vascular dysfunction, ECM alterations and glial activation—captured by the brain and artery aging clocks—collectively contribute to early cognitive decline and NDs during biological aging (Fig. 4g).

**Extended Data Fig. 7:**
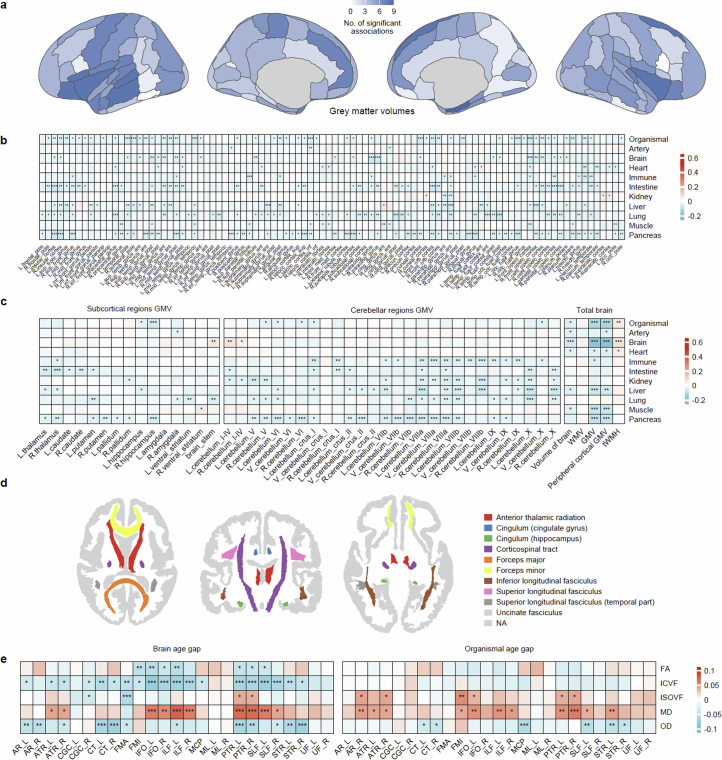
Association of proteomic organ aging clocks with brain structures. **a**, Association of proteomic aging clocks with grey matter volume of cortical regions. Number of significant associations with organ aging clocks were colored for each brain cortical region. **b**, Association of proteomic aging clock with cortical regions GMV. **c**, Association of proteomic aging clock with subcortical regions GMV, cerebellar regions GMV, and summary image-derived phenotypes (volume of brain, WMV, GMV, and tWMH). **d**, White matter tracts of interest. **e**, Association of brain and organismal aging clock with white matter tracts. Generally, brain and organismal aging clock were positively associated with MD and ISOVF, with brain aging clock negatively associated with FA, ICVF, and OD. Associations with brain structures in **b**, **c**, and **e** were estimated by linear regression, presented as beta coefficients. All regression models were adjusted for age, sex, ethnicity, Townsend deprivation index, physical activity level, and recruitment center. All statistical tests are two-sided. FDR using the Benjamini-Hochberg method was used to correct for multiple comparisons. The asterisks denote FDR-adjusted P value thresholds: *q < 0.05; **q < 0.01; ***q < 0.001. FA, fractional anisotropy; ICVF, intracellular volume fraction; ISOVF, isotropic volume fraction; MD, mean diffusivity; OD, orientation dispersion; L, right; R, left; AR, acoustic radiation; ATR, anterior thalamic radiation; CGC, cingulate gyrus part of cingulum; CGH, parahippocampal part of cingulum; CST, corticospinal tract; FMA, forceps major; FMI, forceps minor; IFO, inferior fronto-occipital fasciculus; ILF, inferior longitudinal fasciculus; MCP, middle cerebellar peduncle; ML, medial lemniscus; PTR, posterior thalamic radiation; SLF, superior longitudinal fasciculus; STR, superior thalamic radiation; UNC, uncinate fasciculus.

After identifying the molecular links between brain/body aging and AD, we next examined whether organ aging was associated with structural brain changes over time, as both biological aging and neurodegenerative processes contribute to progressive cerebral atrophy and changes in brain structures (Extended Data Fig. 7). Overall, both brain and organismal aging were significantly associated with reduced total brain volume, decreased total gray matter volume (GMV) and increased total white matter hyperintensity (tWMH). Organ aging was further linked to lower cortical GMV, reduced GMV in multiple subcortical and cerebellar regions, and widespread alterations in white matter microstructure indices across major white matter tracts (Supplementary Note 3). These patterns closely mirror the structural brain changes previously reported in frailty-based biological aging^48^, AD and neurological impairments^49^. Taken together, these findings suggest that structural brain alterations may partially mediate the relationship between biological aging and ND risk.

**Fig. 5:**
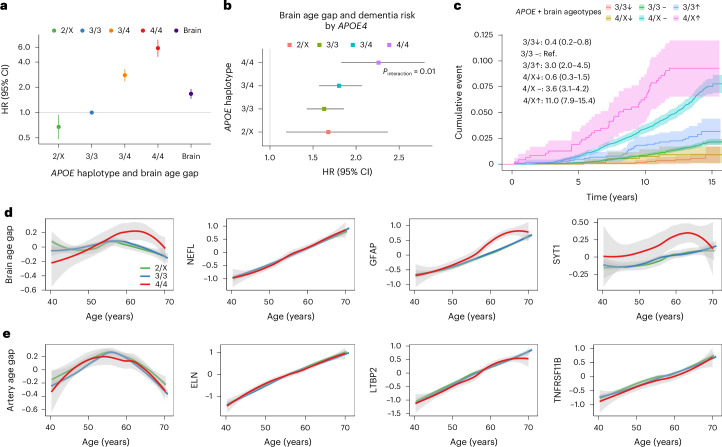
Brain aging and dementia risk across APOE haplotypes. **a**, Associations of *APOE* haplotypes (2/X, 3/3 (reference), 3/4 and 4/4) and the brain age gap with incident dementia (*n* = 29,634). Multivariable-adjusted HRs were estimated using Cox models. **b**, The association between brain aging and dementia was most pronounced among *APOE4* homozygotes, as assessed using Cox models (*P* for interaction = 0.01; *n* = 29,634). Shown are the multivariable-adjusted HRs per 1-s.d. increase in the brain age gap, stratified by *APOE* haplotypes. Squares/circles represent HRs, and error bars indicate the corresponding 95% CIs in **a** and **b**. **c**, Cumulative incidence curves of dementia across joint categories of *APOE* haplotypes (3/3, 4/X) and brain ageotypes (super-youthful (↓), normal (–) and extremely aged (↑)). Compared to participants with *APOE* 3/3 and normal brain aging, *APOE4* carriers with normal and extremely aged brains were at 3.6 and 11.0 times increased risk of dementia, respectively; those with *APOE* 3/3 and a super-youthful or extremely aged brain were at a 60% lower risk and three times increased risk, respectively. **d**, Association of age with the brain age gap and featured component proteins across *APOE* haplotypes. Among *APOE4* homozygotes, a steep rise in the brain age gap and elevated levels of proteins implicated in AD pathology were observed between ages 55 and 65 years—approximately 5–10 years before the average age of dementia onset. **e**, Association of age with the artery age gap and featured component proteins across *APOE* haplotypes. No notable genotype-specific differences in age trajectories were observed (*P* for interaction = 0.16). Trajectories were fitted using Loess regression. The shading around the plotted lines in **c**–**e** indicates the 95% CI. All models were adjusted for age, sex, ethnicity, Townsend deprivation index and recruitment center.

Given that *APOE4* homozygosity has been recognized as a distinct genetic form of AD^50^, we evaluated the performance of the brain aging clock across *APOE* haplotypes and the interactions between the aging clock and *APOE* genotype (Fig. 5). The brain age gap predicted future dementia independently of APOE haplotypes, which themselves were strongly associated with dementia risk in a dose-dependent manner. In a model that included brain age gap, APOE genotypes and their interaction terms, we observed a significant interaction between *APOE4* homozygosity (ε4/ε4) and the brain age gap (*P* = 0.01) (Fig. 5a). The association between brain aging and dementia was more pronounced among *APOE4* homozygotes (Fig. 5b). We next estimated the relative risk of dementia associated with brain ageotypes, stratified by *APOE* ε4 carriers versus *APOE* ε3/ε3 carriers (Fig. 5c). Compared to participants with *APOE* 3/3 and a normally aged brain, *APOE4* carriers with a normally aged brain and those with an extremely aged brain were at 3.6 and 11.0 times increased risk, respectively. Among *APOE* 3/3 carriers, those with super-youthful brains were at a 60% lower risk, while those with extremely aged brains had a threefold increased risk. The dose–response associations between chronological age and the brain age gap varied across *APOE* genotypes (4/4, 3/3 and ε2 carriers). Among *APOE4* homozygotes, a pronounced upward trend in the brain age gap emerged between ages 55 and 65 years—approximately 5–10 years before the average age of dementia onset in this population (median onset age: 76 years for 3/3 and 75 years for 4/4) (Fig. 5d). This elevated trajectory aligns with the time window during which amyloid and tau pathology biomarkers are known to begin rising in *APOE4* homozygotes^50^. Notably, two constituent proteins contributing to the brain age gap—GFAP and SYT1—exhibited similarly divergent dose–response patterns by genotype, a pattern not observed for other proteins or the artery age gap (Fig. 5e). These findings further support the potential utility of the brain aging clock in predicting and stratifying dementia risk across different genetic backgrounds.

**Extended Data Fig. 8:**
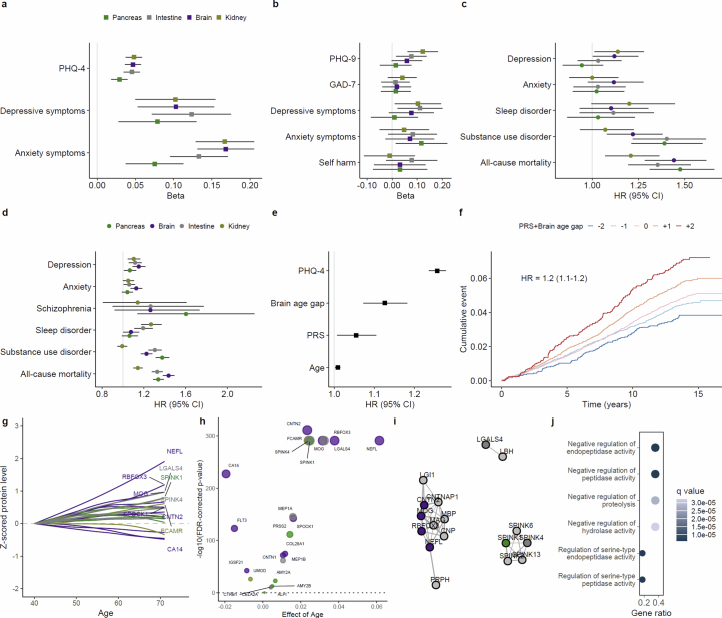
Organismal and brain aging in mental well-being and psychiatric diseases. **a**, Associations of brain and peripheral organ (kidney, intestine, and pancreas) aging (age gaps) with PHQ-4 score and depressive and anxiety symptoms at baseline in participants without neurodegenerative and psychiatric diseases (*n* = 37,764). Associations were estimated by linear regression, presented as beta coefficients. **b**, Associations of brain and peripheral organ aging with future mental health conditions at follow-up in participants without neurodegenerative and psychiatric diseases (*n* = 11,504). **c**, Associations of brain and peripheral organ aging with risk of incident psychiatric diseases and all-cause mortality in participants with psychological distress (symptoms of depression and anxiety) at baseline over 13 years follow-up (*n* = 3,958). **d**, Associations of brain and peripheral organ aging with incident NDs and all-cause mortality in healthy participants over 13 years follow-up (*n* = 43,616). **e**, Association between multiple markers (brain age gap, PRS for depression, and age) and risk of incident depression over 13 years follow-up (*n* = 43,616). Squares represent effect sizes (beta coefficients, ORs, or HRs) and error bars the corresponding 95% CIs in **a**-**e**. **f**, Cumulative incidence curves of depression across combined levels of brain age gap and PRS for depression. Participants were grouped into five bins based on the combined standardized scores: bin -2 (< -1.5 SD), bin -1 (-1.5 to -0.5 SD), bin 0 (-0.5 to +0.5 SD), bin +1 ( + 0.5 to +1.5 SD), and bin +2 (> +1.5 SD). Displayed HR reflects the depression risk per 1 SD increase in the combined scores. **g**, Change of top proteins in organ-specific (brain, kidney, intestine, and pancreas) aging clock that were also related to depression/anxiety with chronological age. Trajectories with age were fitted by Loess regression. Proteins in organismal model are preferentially colored as organ-specific if they are also included in organ-specific aging clocks. **h**, Effect of age on proteins in **g**. **i**, Protein-protein interaction network identified through STRING analysis. Displayed are interactions of the featured proteins from **g** and **h**, and their interacting proteins with a confidence score ≥ 0.4. **j**, Enriched biological pathways for featured proteins involved in aging and depression/anxiety. All relevant models were adjusted for age, sex, ethnicity, Townsend deprivation index, smoking, physical activity level, and recruitment center. Error bars indicate 95% CIs.

Finally, we examined the role of proteomic aging and dysfunction in mental well-being and psychiatric diseases (Extended Data Fig. 8). We identified several proteins associated with psychiatric conditions, including NEFL^51^ and RBFOX3 (refs. ^52,53^); multiple proteins encoded by oligodendrocyte lineage-related genes essential for myelination and myelin structure (MOG, CNP, MAG and MBP)^54,55^; and a protein network within the SPINK family^56^ (Supplementary Note 4). These findings align with mechanistic hypotheses for major depression, such as impaired neurogenesis and neuroplasticity, highlighting the involvement of oligodendrocyte lineage cells in maintaining myelin integrity and synaptic transmission^54,57^. Despite a shared genetic and molecular basis across major psychiatric disorders and NDs, the largely distinct pathogenic pathways associated with differential aging of organs suggest their potential specificity in predicting diseases with overlapping biological mechanisms.

### Predictive performance of the original and refined organ aging clocks versus clinical biomarkers

**Fig. 6:**
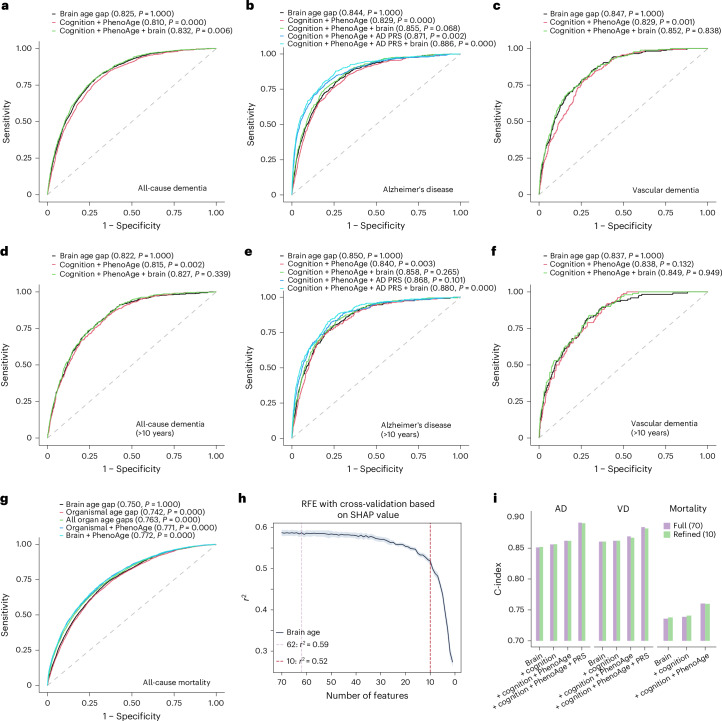
Comparison of predictive performance between proteomic aging clocks (brain and organismal) and clinical biomarkers for dementia and mortality. **a**–**f**, Inclusion of proteomic aging clocks modestly improved risk prediction for all incident cases of ACD (**a**), AD (**b**) and VD (**c**), as well as for >10-year incident cases of ACD (**d**), AD (**e**) and VD (**f**). **g**, Receiver operating characteristic curve analyses for all-cause mortality. Receiver operating characteristic curve analyses based on logistic models were conducted to compare models incorporating basic demographic variables (age, sex and education) and aging clocks, with and without traditional clinical biomarkers. *P* values indicate the significance of differences in predictive performance with the model that includes the brain age gap, age, sex and education, estimated using 2,000 bootstrap iterations. **h**, Refined brain aging clocks using a reduced number of proteins selected using RFE. RFE was performed using SHAP values, with models iteratively fitted using fivefold cross-validation, reducing the number of proteins from 70 to 10. The shading around the plotted lines indicates the 95% CI. **i**, Predictive performance of the refined brain aging clock for dementia and mortality compared to that of the original model. Shown are concordance index (C-index) values across models.

We evaluated the performance of the brain aging clock in predicting dementia and its subtypes, both independently and in combination with other measures, including cognitive function, PhenoAge (based on multiple clinical biomarkers) and PRS (when applicable), while adjusting for basic demographic measures (age, sex and education) (Fig. 6a–f and Supplementary Fig. 4). For incident ACD, AD and VD during the full follow-up period (Fig. 6a–c), the brain age gap demonstrated slightly stronger performance than models including PhenoAge and cognitive function (area under the curve (AUC) 0.844 versus 0.829, bootstrap test *P* < 0.001 for AD; AUC 0.847 versus 0.829, *P* = 0.001 for VD). Adding the brain age gap to models that included clinical and AD PRS further improved predictive power, yielding the strongest performance (Fig. 6b). Similar performance patterns were observed when the modeling was repeated for >10-year incident dementia (Fig. 6e,f). For all-cause mortality, the predictive performance of the brain age gap alone (AUC 0.750) was comparable to that of models incorporating all organ age gaps (AUC 0.763) or both the brain age gap and PhenoAge (AUC 0.772) (Fig. 6g). These findings suggest that the brain aging clock captures key clinical biomarker signals relevant to dementia while providing additional predictive value beyond established clinical and genetic biomarkers.

**Extended Data Fig. 9:**
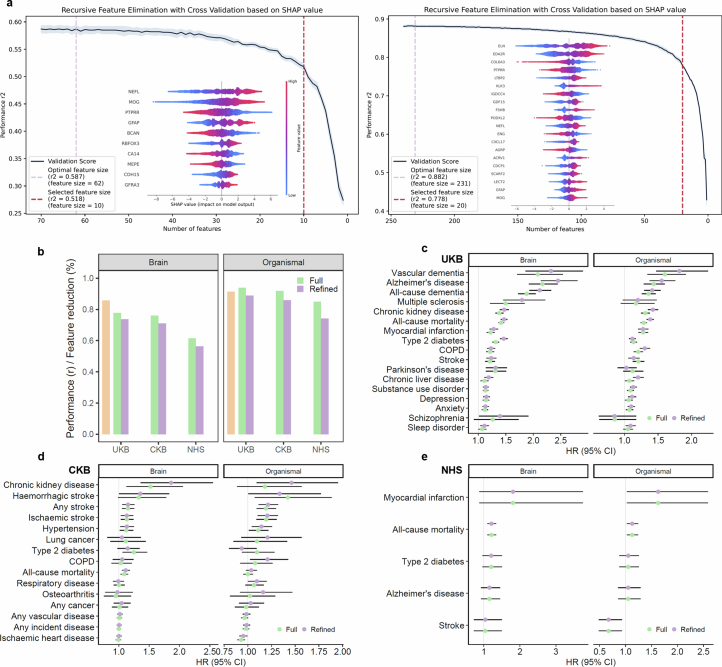
Performance of refined brain and organismal aging clocks with reduced number of proteins and their association with diseases and mortality. **a**, RFE using SHAP values for brain and organismal aging clocks. Models were fitted iteratively using 5-fold cross-validation starting from full protein panels (230 for organismal aging and 70 for brain aging) down to a single protein. At each step, the protein with the smallest absolute mean SHAP value across folds was removed. The R^2^ for explained variance in chronological age was reported as the average across the five folds. Refined brain clocks with 10 proteins and organismal clocks with 20 proteins were ultimately identified. SHAP values for the refined protein panel are shown. **b**, Comparison of the predictive performance for chronological age between the full and refined versions of the brain/organismal aging clocks across cohorts. The orange bar denotes the percentage of feature reduction. **c-e**, Comparison of the association of the full versus refined versions of aging clocks with diseases and mortality in the UKB (*n* = 43,616), CKB (*n* = 3,977), and NHS (*n* = 774). Squares represent HRs and error bars the corresponding 95% CIs. Cox regression models were adjusted for age, sex, ethnicity, Townsend deprivation index, smoking, physical activity level, and recruitment center in UKB; for age, sex, ethnicity, education, study region, smoking, and physical activity in CKB; and for age, ethnicity, neighborhood socioeconomic status, smoking, and physical activity in NHS.

To evaluate translational potential, we developed refined versions of the brain and organismal aging clocks with substantially fewer proteins, selected using recursive feature elimination (RFE) in the UKB (Extended Data Fig. 9a). For the organismal clock, a 20-protein model preserved 88.6% of the original performance (*r*^2^ decreased from 0.88 to 0.78) despite a 92% reduction in protein number (from 240 to 20). Similarly, for the brain clock, a 10-protein model—representing an 86% reduction in protein number (from 70 to 10)—retained 88.4% of the original performance (*r*^2^ decreased from 0.59 to 0.52). These refined clocks maintained their performance in both validation cohorts (Extended Data Fig. 9b). Associations with disease outcomes were largely consistent between the original and refined models across all three cohorts (Extended Data Fig. 9c–e). Notably, the predictive performance of the refined brain aging clock for future dementia was comparable to that of the full model, highlighting its potential clinical utility with only ten nonfasting plasma proteins (Fig. 6h,i).

## Discussion

Leveraging proteomic data from three deeply phenotyped, population-based cohort studies in the UK, China and the USA, we demonstrated that proteomic organ aging clocks provide a robust and interpretable framework for the noninvasive quantification of aging at the organ level. These clocks consistently predict the future risk of disease, multimorbidity and mortality—independently of established clinical biomarkers and risk factors—across diverse populations. Importantly, they reveal distinct organ-specific pathogenic pathways, with the brain aging clock demonstrating particularly strong predictive performance for NDs. In contrast, other physical diseases and mortality are predicted by multiple organ-specific clocks. Among them, the brain aging clock was uniquely associated with the incidence of nearly all major diseases and multimorbidity, independent of other organ clocks, and it showed the strongest association with mortality. Furthermore, sparsified models using only 10–20 nonfasting proteins retained high predictive performance across cohorts, highlighting their clinical utility and feasibility. Collectively, these proteomic clocks offer a promising avenue for informing organ-specific longevity interventions aimed at modulating key proteins and pathways to reduce the burden of age-related diseases and promote healthy aging^5^.

Our proteomic organ-specific aging clocks offer unique and interpretable molecular and cellular insights into organ aging and disease—insights that are largely inaccessible with previous phenotypic or DNAm clocks due to methodological limitations^4^. Notably, our aging clocks showed only weak correlations with established phenotypic clocks for systemic aging, such as PhenoAge^58^ and KDMAge (Klemera–Doubal method age)^59^. In contrast to the largest phenotypic organ-specific clock study using the same UKB data—which failed to associate the magnetic resonance imaging (MRI)-based brain age gap with mortality despite its strong correlation with chronological age^9^—our brain aging clock was a robust predictor of mortality, consistent with findings from another proteomic clock study^3^. Compared to DNAm clocks, our proteomic clocks also offer additional advantages by directly modeling for age, rather than intermediate aging markers, to robustly predict disease and death^60^. DNAm clocks trained solely on age often perform poorly in predicting health outcomes, potentially due to weak correlations between gene expression and protein levels, as well as the disconnect between DNAm changes and functional protein activity^6^. For example, key proteins prioritized by our models—such as ECM proteins (for example, ELN) involved in vascular aging and inflammatory markers (for example, GFAP)—are unlikely to be captured by DNAm clocks due to their long half-lives, post-translational modifications and weak associations with transcriptomic data. These findings highlight the superiority of data-driven, organ-specific protein biomarkers over manually selected clinical measures, which tend to reflect systemic aging and disease burden rather than localized biological aging. Compared to existing proteomic organ aging clocks, about half of the APs in our Olink-based clocks were not identified in SomaScan-based clocks^3,16^, suggesting the potential complementarity of different proteomic platforms. Despite representing a largely distinct set of APs, several top proteins in our clocks—such as NEFL and CNTN2 for brain aging—were also featured in blood- and cerebrospinal fluid-based SomaScan brain clocks^3,61^, supporting the biological relevance and generalizability of our findings. Notably, in contrast to SomaScan clocks that include hundreds of proteins for major organs, our study further demonstrates that this framework can be extended to sparse models with as few as ten proteins per organ, many of which are already used as response markers in clinical trials^34,35,62^, underscoring its potential clinical utility and feasibility.

Our models identified largely distinct proteins and pathways associated with NDs and psychiatric diseases, despite their shared genetic structures and risk factors^63^. Specifically, the artery and brain aging clocks captured key biological processes implicated in the pathogenesis of AD and VD, including synaptic and neuronal degradation, glial activation, vascular dysfunction and ECM remodeling^41–43,46,47^. Key proteins in the brain aging clock, such as NEFL^35^, GFAP^34^, SYT1 (ref. ^38^) and CNTN1 (ref. ^64^), are genetically and biologically implicated in neurological disorders, supporting their potential causal role in brain aging. Notably, NEFL has recently been approved as a surrogate endpoint for ND drug trials^62^. While response to intervention is essential for aging biomarkers, few composite response markers have been discovered to date^5^. Our findings highlight the potential of proteomic organ aging clocks to serve as candidate biomarkers for monitoring responses to interventions targeting diseases and promoting longevity.

Furthermore, we systematically characterized both genetic and environmental determinants of organ aging, revealing links between proteomic age acceleration and structural brain changes. For example, we identified *GABBR1*, which encodes the GABA-B receptor, as a top genetic signal for brain aging. This gene is involved in synaptic signaling and represents a promising therapeutic target for cognitive and neuropsychiatric disorders^17–20^. The shared genetic architecture of biological aging and age-related diseases supports the geroscience concept of targeting aging itself to treat multiple diseases and extend healthspan^65^. The broad tissue expression of brain aging-associated genes, consistent with previous GWASs^31,66^, further suggests that brain aging is regulated by physiological processes across both the brain and peripheral systems. In addition, we identified that adherence to a healthy lifestyle—including regular physical activity and sufficient sleep—was associated with decelerated aging of multiple organs, particularly the brain, intestine and pancreas. This aligns with recent causal evidence linking modifiable lifestyle factors such as sleep duration and body weight to phenotypic organ-specific aging^31^. Overall, these models may be further leveraged in human intervention studies—such as those focused on lifestyle modifications or pharmacological therapies—to track longitudinal responses and gain mechanistic insights into the biology of healthy aging.

This study has several limitations. First, while our clocks demonstrated robust external validity across populations with diverse genetic and environmental backgrounds, their reliance on relative protein quantification warrants further validation using absolute measurements, especially for the brain aging clock. Second, while the refined clocks with fewer proteins preserved their performance across cohorts, the excluded proteins may still hold biological relevance. Thus, the full clocks enable a more detailed molecular interpretation, whereas the refined versions demonstrate potential translational utility. Third, despite reliably predicting diseases and aging phenotypes across cohorts, some organ clocks exhibit only weak to modest correlations with chronological age (*r* = 0.3–0.5). While statistically robust correlations with chronological age are necessary, their optimal strength remains uncertain and likely varies across organ systems. A model that perfectly predicts age would add little biological insight. The observed modest correlations—consistent with previous organ clocks and aging models—may, in fact, be more informative for capturing interindividual variation in biological aging. Future studies with larger sample sizes and more sophisticated modeling strategies may further optimize prediction accuracy while preserving biological interpretability. Fourth, although predictive performance was largely replicated in the NHS, the correlations with age were weaker than in the UKB and CKB, likely reflecting the smaller sample size and warranting further validation in larger, more diverse populations. Fifth, our models were based on approximately 3,000 proteins from the Olink 3072 assay, which spans ten organ systems. Therefore, they do not capture the full plasma proteome or all organs, underscoring the need for broader panels and organ coverage. Sixth, our stringent definition of organ-enriched proteins may have limited the inclusion of informative, broadly expressed proteins that could further enhance prediction accuracy. Loosening this criterion may increase sensitivity at the expense of organ specificity—a trade-off that merits further investigation. Seventh, the causality of the APs identified, as well as the relationship between a healthy lifestyle and younger organs, should be further assessed to inform potential longevity interventions. Finally, although we demonstrated the distinct added value of current proteomic models over previous phenotypic or DNAm organ aging models, direct comparisons to other organ-specific models remain necessary to contextualize their added value.

In summary, this study represents the most comprehensive evidence to date of the biological and clinical utility of proteomic aging clocks for the noninvasive quantification of aging at both organ-specific and systemic levels. Our models demonstrate superior performance in predicting disease risk and tracking longevity compared to established clinical and genetic biomarkers, while uncovering distinct pathogenic pathways and potentially modifiable targets. Importantly, we also identified parsimonious protein panels that retain high predictive accuracy and are well-suited for clinical translation. By revealing the proteomic convergence of biological aging and disease across organ systems, these aging clocks provide a robust and interpretable framework to guide targeted, organ-specific interventions aimed at reducing the burden of age-related diseases and promoting healthy aging.

## Methods

### Study populations

All contributing cohorts (UKB, CKB and NHS) received ethical approval from their respective institutional review boards, and all participants consented to the use of their anonymized information for research purposes at the time of recruitment. All participants from the UKB and CKB provided written informed consent. In the NHS, institutional review boards approved questionnaire completion as implied consent.

The UKB is a prospective population-based cohort of more than 500,000 individuals aged 40–70 years who were recruited between 2006 and 2010 from the UK general population, with deep phenotyping and genomic data available^67^. Participants were followed up through data linkage to electronic health and medical records, including national primary and secondary care, as well as disease and mortality registries^68^, with validated reliability, accuracy and completeness^69^. Additional online surveys were conducted to facilitate the follow-up of cognitive and symptom-based mental well-being outcomes. In the current study, we included a subset of randomly selected, representative UKB participants with Olink proteomics data available at baseline (*n* = 46,785).

The CKB is a prospective cohort study of 512,724 adults aged 30–79 years who were recruited from ten geographically diverse (five rural and five urban) areas across China during 2004 to 2008 (ref. ^70^). We included CKB participants with baseline Olink data in a nested case–cohort study of ischemic heart disease and who were not genetically related (*n* = 3,977).

The NHS is a prospective cohort study involving 121,700 female registered nurses from 11 US states, aged 30–55 years at enrollment in 1976, with follow-up data collected using biennial questionnaires^71^. Between 1989 and 1990, a total of 32,825 participants provided blood samples. We included NHS participants with Olink data in a prospectively designed nested case–cohort study of colon cancer (*n* = 800).

### Proteomic profiling

Proteomic profiling of baseline blood plasma samples was conducted for all three cohorts using the same Olink Explore 3072 assay, which includes four panels (cardiometabolic, inflammation, neurology and oncology) measuring 2,923 independent proteins. Among 54,219 UKB participants with available Olink data, we included 46,673 individuals who were randomly selected and shown to be highly representative of the broader UKB population^72^, excluding those manually selected for disease enrichment. In the UKB, no effects of batch and plate, or abnormalities in the protein coefficients of variation, were observed. The interplate and intraplate coefficients of variation for all Olink panels were lower than 20% and 10%, respectively, with a median of 6.7% (ref. ^72^). High correlations were observed for the same proteins across panels and between the Olink assay and independent assays. Details of Olink proteomic measurements, data processing and quality control in the UKB are described in the online document (https://biobank.ndph.ox.ac.uk/showcase/label.cgi?id=1839) and published work^72^. Details of proteomic profiling in the CKB and NHS are provided in Supplementary Notes 5 and 6. Proteomics data across all cohorts were provided as normalized protein expression values on a log_2_ scale. We excluded seven proteins that were missing in more than 20% of UKB participants (GLIPR1, NPM1, PCOLCE, CST1, CTSS, TACSTD2 and ENDOU). Participants with more than 50% missing proteins were further excluded. The final UKB dataset included 43,616 participants and 2,916 proteins. Normalized protein expression data were rescaled to range between 0 and 1 and then centered on the median.

### Organ-specific protein mapping

Organ-enriched genes and plasma proteins were determined using human organ bulk RNA-seq data from the GTEx project (v8; 54 tissue types)^11^ and were validated using data from the Human Protein Atlas (HPA)^73^ (Extended Data Fig. 1 and Supplementary Tables 1–3). Genes were defined as organ-enriched when their expression was at least fourfold higher in one organ than in any other, following the HPA criteria validated in previous studies^3^. In GTEx, tissues were initially mapped to corresponding organs based on physiological function^3^, and organ-level gene expression was established by identifying the maximum expression value among its tissue subtypes. Identified organ-enriched genes were further tested using the same criteria in the HPA tissue-level data. We annotated the 2,916 plasma proteins measured by the Olink panel with this information.

### Disease, biomarker and age-related phenotypes

In the UKB, the primary disease outcomes are major NDs, such as ACD, VD, AD, PD, MS and psychiatric diseases, including psychotic disorders, mood disorders, anxiety disorders, sleep disorders and substance use disorders. For psychiatric diseases, we assessed the major subtype in each category separately (for example, schizophrenia in psychotic disorders, depression in mood disorders and generalized anxiety disorder in anxiety disorders). Incident disease diagnoses were ascertained using International Classification of Diseases (ICD) codes from linked hospital inpatient, primary care (with read codes transformed into ICD codes) and death registry data. Self-reported cases were not considered to ensure the reliability of the diagnosis, but they were used to identify and exclude participants with relevant prevalent diseases. For comparison, we also included major chronic physical diseases directly relevant to specific organs, including hypertension, myocardial infarction, stroke, chronic obstructive pulmonary disease, CKD, chronic liver disease and T2D. Detailed definitions of disease outcomes are provided in Supplementary Table 12. For additional benchmarking, we included all-cause mortality, which was ascertained through linkage to the national death registry. Details of the mapping process for incident disease outcomes are available online (https://biobank.ctsu.ox.ac.uk/crystal/refer.cgi?id=593).

Other phenotypes of interest included biomarkers (blood chemistry, blood count, nuclear magnetic resonance (NMR) metabolites and neurobiomarkers), as well as age-related physiological, cognitive and mental well-being conditions available among participants with proteomic data. Detailed definitions of these phenotypes are provided in Supplementary Table 13.

Blood chemistry markers and blood counts were measured using nonfasting serum samples from all participants at baseline. Biochemical measures were adjusted for technical variation, with details of sample processing (https://biobank.ndph.ox.ac.uk/showcase/showcase/docs/serum_biochemistry.pdf) and quality control (https://biobank.ndph.ox.ac.uk/showcase/ukb/docs/biomarker_issues.pdf) provided online. A total of 30 biochemistry markers (related to liver and renal function, endocrine status and immunometabolism) and 31 blood cell counts (including white blood cells, red blood cells and platelets) were used.

NMR metabolites were measured using baseline plasma samples from approximately one third of randomly selected participants (*n* = 118,461) in the UKB, including absolute concentrations of 168 biomarkers (81% are lipids and lipoprotein subfractions) along with 81 ratios of these biomarkers. Details of sample processing and quality control are available online (https://biobank.ndph.ox.ac.uk/showcase/label.cgi?id=220). We included only nonratio NMR measures in the current analysis.

Plasma neurobiomarkers were measured for a subset of participants (*n* = 1,268) who engaged in the first brain imaging visit (2014 and thereafter), including plasma Aβ40, Aβ42, GFAP, NEFL and pTau-181.

Age-related physiological phenotypes measured at baseline included self-rated health (poor versus others), usual walking speed, self-rated facial aging (older than you are versus others), tiredness and lethargy (nearly every day versus others), sleeplessness (usually versus others), hand grip strength (standardized by weight), systolic and diastolic blood pressure (average of multiple readings), body mass index, lung function (forced expiratory volume in 1 s standardized by height squared) and leukocyte telomere length (T/S ratio—ratio of the telomere repeat copy number (T) to the copy number of the single-copy gene *HBB* (S)—corrected for technical variation, log-transformed and *z*-standardized).

Cognition and mental well-being outcomes were measured at baseline and follow-up surveys using questionnaires. Cognitive function phenotypes included reaction time (a measure of processing speed, which is a component of general cognitive function), numerical memory (a measure of numerical short-term memory), fluid intelligence (a score that assesses crystallized and fluid intelligence in both verbal and numerical aspects) and visual memory (a measure of visuospatial working memory). These four tests demonstrated high validity and reliability^74^, as well as predictive ability for incident dementia in the UKB. Reaction time was available for all participants at baseline and was phenotypically and genetically related to general cognitive function, which was then used as a major cognitive function phenotype. The last three cognition measures were available for a subset of participants at baseline and were tested in the online follow-up surveys (2014 and thereafter and 2021 and thereafter). Mental health and well-being outcomes included the Patient Health Questionnaire 4 (PHQ-4), which measures the general symptoms of depression and anxiety; self-rated health at baseline; the PHQ-9, which measures the severity of depressive symptoms; the Generalized Anxiety Disorder 7 Scale (GAD-7), which measures generalized anxiety symptoms; self-harm behavior, including self-harm and suicidal ideation/behavior; mental distress; happiness; satisfaction with one’s own health; and self-rated health from the online follow-up survey. Other general health outcomes included self-rated health at baseline and imaging visits. Details of the online cognition and mental well-being survey are available online (https://biobank.ndph.ox.ac.uk/showcase/refer.cgi?id=2800).

Modifiable lifestyle factors included smoking, alcohol consumption, physical activity, sedentary time, sleep duration, intake of fruits and vegetables, intake of oily fish, intake of red meat and intake of processed meat. Detailed definitions and classifications of lifestyle factors are provided elsewhere^75^.

In the CKB, incident diseases and cause-specific mortality were ascertained through electronic linkage to national registries and health insurance records^70^. All disease diagnoses were coded using ICD-10, with baseline information kept blinded. Participants were followed until death, loss to follow-up (<1%) or 1 January 2019. Detailed definitions of disease outcomes are provided in Supplementary Table 14.

In the NHS, deaths were ascertained through state vital records, the National Death Index, next of kin and postal authorities. Incident cases of cancer, myocardial infarction and stroke were initially self-reported on biennial questionnaires and subsequently confirmed through a physician review of medical records. Self-reported diagnoses of incident T2D were validated using a supplementary questionnaire. Dementia cases were identified based on physician-reviewed death records and biennial self-reported physician diagnoses of AD or other dementias.

### Brain MRI data

All brain MRI data in the UKB were acquired using a 3-T Siemens Skyra scanner, preprocessed with quality control and summarized as image-derived phenotypes (IDPs). We used the data (*n* = 49,002) from the first brain imaging visit (2014 and thereafter). Details of image acquisition, processing, quality control and phenotype calculation are described in the online document (https://biobank.ctsu.ox.ac.uk/showcase/showcase/docs/brain_mri.pdf) and published work^76^. Tissue type and gray matter segmentation of magnetic resonance images was performed using FAST (FMRIB’s Automated Segmentation Tool), while subcortical structures were modeled using FIRST (FMRIB’s Integrated Registration and Segmentation Tool). The GMVs of 139 cortical, subcortical and cerebellar regions based on the Harvard–Oxford atlas and the Diedrichsen cerebellar atlas were then derived from T1-weighted MRI. The tWMH and microstructural measures of white matter tracts (fractional anisotropy, mean diffusivity, intracellular volume fraction, orientation dispersion and isotropic volume fraction) were derived from diffusion MRI. Other IDPs, such as total brain volume and subcortical volume, were also included. Extreme outliers (outside ±4 s.d.) that may reflect processing errors or brain abnormalities were excluded on a case-wise basis (<0.001% of IDP data points analyzed). WMHs were log-transformed to normalize the positively skewed distribution. IDPs were further adjusted for head size by multiplying the raw volumes by the volumetric scaling factor.

### Polygenic risk score

PRSs were generated by the UKB using a Bayesian approach applied to summary statistics of external (ancestry-specific, when applicable) GWAS meta-analysis with no sample overlap with the UKB population, as described online (https://biobank.ndph.ox.ac.uk/showcase/refer.cgi?id=5202). We extracted PRSs for AD, PD, schizophrenia, MS and bipolar disorder.

### Calculation of biological age gap and extreme ageotypes

Chronological age as a decimal value was calculated by taking the number of days between the baseline assessment date and the approximate birth date (based on the month and year of birth, with the first day of the birth month assigned as the birth date) and dividing that number by 365.25 in all three cohorts.

All eligible UKB participants (*n* = 43,616) were randomly split into training and testing sets with a 7:3 ratio. To identify APs and estimate proteomic age, we used the LightGBM^77^ model, which outperformed five other alternative machine learning models, including LASSO, Elastic Net and three artificial neural network architectures (multilayer perceptron, ResNet and TabR), in predicting overall organismal proteomic age (Supplementary Note 7)^7^. Sex-specific models for organismal aging showed high correlations with the overall model for both sexes (*r* = 0.99 and 0.98, respectively), supporting the use of combined-sex models to enhance the generalizability of findings.

In the UKB training set (*n* = 30,536), using LightGBM, we trained one organismal aging model based on all 2,916 proteins and ten organ-specific aging models based on the selected proteins enriched in the brain, heart, lung, immune system, artery, intestine, liver, muscle or pancreas (Supplementary Table 3). First, we tuned the model hyperparameters through fivefold cross-validation using the Optuna module in Python^78^. Across 200 trials, parameters were tested and optimized to maximize the average model *R*^2^ across all folds. Second, we performed Boruta feature selection using the shap-hypetune module, which randomly permutes all model features (referred to as shadow features, representing random noise)^79^ and helps distinguish the true signal from noise. Shadow features were generated at each iteration, and a model was trained using all features and the shadow features. Features were then evaluated based on their mean Shapley Additive Explanations (SHAP) values, a measure of feature importance. Features with absolute mean SHAP values lower than those of all random shadow features were removed. We conducted Boruta selection with 200 trials, setting a 100% threshold to compare shadow features and real features. Third, we retuned the model hyperparameters for a new model based on the selected protein subset, using the same procedure as above. Both tuned LightGBM models—before and after feature selection—were evaluated for overfitting and validated using fivefold cross-validation on the combined training set, followed by performance testing on the independent holdout test set. The holdout testing set was reserved during parameter tuning and feature selection and was used only for performance evaluation.

Based on the final trained model with Boruta-selected APs, we calculated organismal and organ-specific proteomic age for the full UKB sample (*n* = 43,616) using fivefold cross-validation. Within each fold, predicted age values were calculated by training a LightGBM model using the final hyperparameters, which were then aggregated across folds to generate the proteomic age for the full sample. Finally, based on proteomic age, we defined two aging phenotypes: age gap (the residual of proteomic age regressed on chronological age, reflecting accelerated or delayed aging compared to same-aged peers) and extreme ageotypes (a 1.5 s.d. increase or decrease in at least one organ age gap, reflecting individuals with extremely aged or youthful organs).

Given the prior knowledge that hundreds of proteins are related to organismal and organ aging^3^, we further refined the selection of associated proteins through RFE using SHAP. This process aims to identify the minimum number of proteins necessary for the accurate prediction of aging. In the RFE analysis, we started with the full set of APs identified by Boruta. We trained models using fivefold cross-validation on the training set and calculated the mean *R*^2^ and absolute SHAP values for each protein across the folds. We then iteratively eliminated the protein with the lowest SHAP value at each step and trained a new model until the final model included only one protein. We recalculated proteomic age and aging phenotypes based on the refined proteins using the same methods as described above, with the number of proteins determined by visual inspection of *R*^2^ following RFE. We compared the association of outcomes between the refined aging clock with decreasing number of proteins and the original clock that uses the full set of Boruta-selected proteins.

Proteomic organ age in the CKB and NHS was predicted using the trained UKB model (full or RFE-refined panel), and the organ age gap was calculated in these datasets following the same approach as in the UKB.

For comparison, we also measured biological age based on clinical traits using the validated KDM-biological age (KDM-BA)^59^ and PhenoAge^58^ algorithms, which predict the risk of death and morbidity. These aging measures were trained using data from the National Health and Nutrition Examination Survey and projected onto the UKB data (Supplementary Note 8). PhenoAge was calculated based on albumin, alkaline phosphatase, C-reactive protein and glucose levels, along with lymphocyte proportion, mean cell volume, white blood cell count and red cell distribution width. The KDM-BA was calculated based on albumin, alkaline phosphatase, C-reactive protein, creatinine, glycated hemoglobin, total cholesterol, blood urea nitrogen and systolic blood pressure levels.

### Missing data imputation

Missing values for all covariates, except age, in the UKB were imputed using the R package missRanger, which combines random forest imputation with predictive mean matching. Proteomic data were not imputed, leveraging the default capability of LightGBM to handle missing values. During model training, missing values for numerical features were assigned to the side of the ongoing split that yields the greatest reduction in loss, maximizing the information gain within the decision tree construction process without necessitating imputation. The imputation dataset comprised 100 baseline variables across multiple domains (physical health, environment and lifestyle) as predictors for imputation, excluding variables with nested response patterns. Responses of ‘do not know’ were treated as missing values (NA) and imputed. However, responses of ‘prefer not to answer’ were not imputed and were set to NA in the final analysis dataset. Study outcomes were not imputed. CKB and NHS data had no missing values for imputation.

### Statistical analysis

Associations of age gap or extreme ageotypes (presence and number) across organ systems with incident diseases, multimorbidity and all-cause mortality in the UKB were assessed using Cox proportional hazards regression models, adjusting for age at recruitment, sex, ethnicity, education level, Townsend deprivation index, International Physical Activity Questionnaire activity group, smoking status and recruitment center. The follow-up period commenced from the date of baseline recruitment to the earliest date of disease diagnosis from the three data sources, the date of death, or the last available date provided by the hospital or general practitioner, whichever came first. Participants with a history of the corresponding outcome before the start of follow-up were excluded. The proportional hazards assumption was tested using Schoenfeld residuals, with no violations observed. The FDR calculated using the Benjamini–Hochberg method was applied to correct for multiple comparisons. Kaplan–Meier plots were generated to depict the cumulative incidence of outcomes over time. To further evaluate the potential clinical utility of organ aging clocks for risk stratification, we tested the association of organ age gaps in combination with other biomarkers using a multivariate Cox model, adjusting for the same covariates.

Associations of organ age gaps and biological aging acceleration (KDM-BA and PhenoAge gaps) with blood chemistry measures, blood counts, neurobiomarkers, age-related physiological conditions, cognition and mental well-being, NMR metabolites, and modifiable lifestyle factors were assessed using linear or logistic regression models, with adjustments for age, sex, education level, Townsend deprivation index, International Physical Activity Questionnaire activity group, smoking status and recruitment center, where applicable. *P* values were corrected for multiple comparisons using the FDR.

To test the role of the organ age gap in the transition of cognitive function and disease in the UKB, we assessed the association of organ age gaps with three sets of longitudinal changing patterns: (1) transition from cognitively normal at baseline (without NDs) to MCI at 12 years of follow-up. MCI at follow-up was defined as a global cognitive score 1.5 s.d. below the baseline mean of cognitively normal participants stratified by educational level^80^. (2) Transition from MCI at baseline to incident NDs at follow-up. MCI at baseline was defined as a global cognitive score 1.5 s.d. lower than the stratified baseline mean score. (3) Transition from psychological distress at baseline (without psychiatric disease) to incident psychiatric diseases at the follow-up. Psychological distress (subclinical symptoms of depression and anxiety) at baseline was defined as a PHQ-4 score of ≥6 (ref. ^81^). Baseline cognitive and mental health conditions were adjusted for in the Cox regression model.

Receiver operating characteristic curve analyses were conducted to compare the predictive performance of the proteomic aging clock to that of clinical and genetic biomarkers for disease and mortality, based on the logistic regression model. We tested the predictive performance of organ aging both independently and in combination with other measures, including cognitive function, PhenoAge and PRS (when applicable), while adjusting for basic demographic measures (age, sex and education). Differences in the AUC between models were assessed using bootstrap tests with 2,000 iterations.

External validation of the association of organ aging with diseases and death in the CKB and NHS was conducted using Cox regression models, as in the UKB. Models were adjusted for age, sex, ethnicity, education, study region, smoking and physical activity in the CKB. In the NHS, models were adjusted for age, ethnicity, neighborhood socioeconomic status, smoking and physical activity. To account for potential bias from the case–control design, analyses were performed on both the full and control samples, with cross-cohort comparisons based on estimates from the control groups of the CKB and NHS.

All analyses and data visualizations were conducted using R (v.4.2) and Python (v.3.6).

### Genotyping, genome-wide association analysis and annotation

DNA extraction, genotyping and initial quality control of the genomic data in the UKB have been detailed elsewhere^67^. Briefly, 488,377 participants were genotyped using two similar genotyping arrays (UK BiLEVE and UKB Axiom Array), and data were imputed using the UK10K reference panel, the 1000 Genomes phase 3 panel and the Haplotype Reference Consortium panel. The GWAS on the proteomic aging clock was conducted using REGENIE, which better accounts for potential population stratification and family relatedness^82^. The regression models were adjusted for age, sex, genetic batch and the first ten genetic PCs. Data were filtered to include SNPs with a minor allele frequency of >1%, a minor allele count of ≥20, an imputation quality INFO score of >0.1 and a Hardy–Weinberg equilibrium value of ≥1 × 10^−15^ for participants with available proteomic data. To further account for population stratification, the current analysis was restricted to participants of European ancestry, as confirmed by the PCs of the genotyped variants.

GWAS summary results were then mapped and annotated using the SNP2GENE module in FUMA v1.5.2. A precalculated linkage disequilibrium structure based on the 1000 Genomes European reference population was used. Independent significant SNPs (*P* < 5 × 10^−8^ and *r*^2^ < 0.6) were first identified, and independent lead SNPs at *r*^2^ < 0.1 were then defined. Closely located linkage disequilibrium blocks of independent significant SNPs (±250 kb) were merged into genomic loci. SNPs in the genomic risk loci were mapped to 19,839 protein-coding genes using the positional mapping (based on ANNOVAR; 10-kb window), eQTL mapping (based on GTEx v8) and chromatin interaction mapping methods.

Gene-based association analysis was conducted using MAGMA. SNPs were mapped to 19,839 protein-coding genes (0-kb window). Gene-based *P* values were calculated using an SNP-wide mean model, with 1000 Genomes phase 3 used as a reference panel. Bonferroni correction was applied to correct for multiple testing (*P* = 0.05/19,839). MAGMA tissue enrichment analysis was then conducted for related genes using gene expression data from 30 general tissues in GTEx v8. Based on these expression data, differentially expressed gene sets were created, and the prioritized genes were tested against these sets to determine whether the genes were upregulated or downregulated in a specific tissue compared to others. Finally, to test the overrepresentation of biological functions, the prioritized genes were evaluated against predefined gene sets such as MSigDB (Molecular Signatures Database), WikiPathways and GWAS Catalog-reported genes.

### Functional enrichment and protein–protein interaction

Functional enrichment analysis based on Gene Ontology (GO) biological processes, GO molecular functions and KEGG (Kyoto Encyclopedia of Genes and Genomes) pathways was performed using all human genes as the background distribution. Enrichment was assessed using the hypergeometric test, and *P* values were adjusted for multiple testing using the Benjamini–Hochberg method. Sensitivity analyses were conducted using only proteins included in the Olink Explore platform as the statistical background. Protein–protein interaction networks were generated using the STRING database^83^.

### Single-cell and bulk RNA sequencing

Preprocessed human scRNA-seq data for the brain^40^ and peripheral vasculature^45^ were accessed from studies in the Human Cell Atlas (https://cellxgene.cziscience.com/gene-expression). Preprocessed human scRNA-seq data for the brain vasculature were accessed from ref. ^44^. Gene expression count data were log(transcripts per million + 1)-transformed and *z*-scored for visualization. Differential expression data of RNA and proteins between AD and control cases were obtained from ref. ^84^

### Reporting summary

Further information on research design is available in the Nature Portfolio Reporting Summary linked to this article.

## Supplementary information

Supplementary Information Supplementary Notes 1–8, Figs. 1–5 and Table descriptions.

Reporting Summary

Supplementary Tables Supplementary Tables 1–18.

## Source data

Source Data Extended Data Fig. 1 Statistical source data.

## Data Availability

Researchers can apply to use the UK Biobank dataset by registering and applying at https://ukbiobank.ac.uk/register-apply/. Use of the UK Biobank was approved by the UK Biobank Ethics Advisory Committee under application 98358. The CKB is a global resource for the investigation of lifestyle, environmental, blood biochemical and genetic factors as determinants of common diseases. The CKB study group is committed to making the cohort data available to the scientific community in China, the UK and worldwide to advance knowledge about the causes, prevention and treatment of disease. For detailed information on what data are currently available to open-access users, how to apply for them and the timeline for data access, visit the CKB website at https://www.ckbiobank.org/data-access. Researchers who are interested in obtaining the raw data that have been officially released from the CKB study should contact ckbaccess@ndph.ox.ac.uk. A research proposal will be requested to ensure that any analysis is performed by bona fide researchers. For any data that are not currently available via open access, researchers may need to develop a formal collaboration with the CKB study group. Because of participant confidentiality and privacy concerns, NHS data are available upon written request. According to standard controlled access procedure, applications to use NHS resources will be reviewed by our External Collaborators Committee for scientific aims, evaluation of the fit of the data for the proposed methodology, and verification that the proposed use meets the guidelines of the Ethics and Governance Framework and the consent that was provided by the participants. Investigators wishing to use NHS data are asked to submit a brief description of the proposed project (contact: nhsaccess@channing.harvard.edu). Investigators can expect initial responses within 4 weeks of request submission, as detailed on https://www.nurseshealthstudy.org/researchers. Human organ bulk RNA sequencing data from the Genotype–Tissue Expression project and Human Protein Atlas are available at https://www.gtexportal.org/home/downloads/adult-gtex/bulk_tissue_expression and https://www.proteinatlas.org/about/download, respectively. Preprocessed human scRNA-seq data for the brain^40^ and peripheral vasculature^45^ were accessed from studies in the Human Cell Atlas (https://cellxgene.cziscience.com/gene-expression). Preprocessed human scRNA-seq data for the brain vasculature were accessed from ref. ^44^. Differential expression data of RNA and proteins between AD and control cases were obtained from ref. ^84^. All additional summary data generated and/or analyzed in the current study are available from the corresponding author on reasonable request. Source data are provided with this paper.

## Code availability

Analysis code used for this study is available on GitHub at https://github.com/41way5/Organ-PAC.
