# Supplementary material for: Organ-specific proteomic aging clocks predict disease and longevity across diverse populations
Source: Nat Aging. 2025 Nov 26;6(1):162–80. doi: 10.1038/s43587-025-01016-8 (PMC12823432; doi:10.1038/s43587-025-01016-8)
Supplement: Supplementary file 1 — Supplementary Notes 1–8, Figs. 1–5 and Table descriptions. [file 43587_2025_1016_MOESM1_ESM.pdf]

nature aging

Supplementary information

https://doi.org/10.1038/s43587-025-01016-8

Organ-specific proteomic aging clocks predict disease and longevity across diverse populations

In the format provided by the authors and unedited

Table of Contents

Supplementary Notes 2

Supplementary Figures 9

Supplementary table descriptions. 16

Supplementary Notes

1. **Clinical biomarkers, age-related traits and organ aging clock**

After adjusting for chronological age, sex, major sociodemographic and lifestyle covariates, all organ and organismal aging were significantly associated with age-related physiological phenotypes (**Fig. 2c**), such as increased levels of BMI, SDP, DBP, sleeplessness, and facial aging, as well as decreased levels of telomere length, walking speed, lung function, and overall health rating. Organismal and organ aging, especially brain, intestine, and pancreas, were related to worse cognitive function and mental health conditions at baseline.

There were also widespread associations with blood biochemistry (**Fig. 2c**). The representative associations with biomarkers that clinically used as specific or general markers of organ function included higher BUN (marker of kidney dysfunction and heart failure; 13/13 proteomic/phenotypic aging clock), lower albumin (a general sign of liver or kidney diseases or worse health; 12/13), higher ALT (a marker of liver damage or diseases of heart and kidney; 10/13 clock; kidney r = 0.24), higher AST (a marker of liver damage or diseases of heart and kidney; 12/13; kidney r = 0.12), creatine (a standard clinical marker of kidney function and health; 13/13; kidney r = 0.21), and CRP (a general marker for inflammation and infection; 9/13; kidney r = 0.17). Organismal and organ aging were associated with NMR-measured metabolites, with organs except for heart related to atherosclerotic lipid profiles (high ApoB, LDL-C, and TG, low HDL-C and ApoA1) and high glucose levels (**Fig. 2d**).

We then assessed the association of modifiable lifestyle factors (smoking, alcohol intake, physical activity, sedentary time, sleep duration, and dietary habits) with organ aging clock (**Fig. 2e**). Almost all unhealthy lifestyle factors were related to organ and organismal aging, with the exception of alcohol and red meat. Frequent alcohol intake was inversely related to aging in artery, intestine, kidney, and lung as well as phenotypic age, but was positively related to pancreas aging. Overall, increasing numbers of unhealthy lifestyle factors were associated with accelerated aging in most organs. Compared to participants with an unfavorable lifestyle (7-10 unhealthy factors), those adhered to a favorable lifestyle (0-4 factors) had significantly lower brain age gap (beta = -0.21), intestine (-0.22), liver (-0.09), lung (-0.12), pancreas (-0.27). These cross-sectional findings suggested that adherence to a healthy lifestyle is related to youthful organ systems, although further prospective investigations are needed to assess the causality and direction of association.

Finally, we assessed the association of brain age gap with neurobiomarkers (Aβ40, Aβ42, GFAP, NEFL, pTau181, Aβ42/Aβ40) measured at follow-up visit and with PRS for AD (n = 124), which are established early biomarkers for neurodegeneration. Although the sample size was limited, brain age gap was largely not explained by these neurobiomarkers.

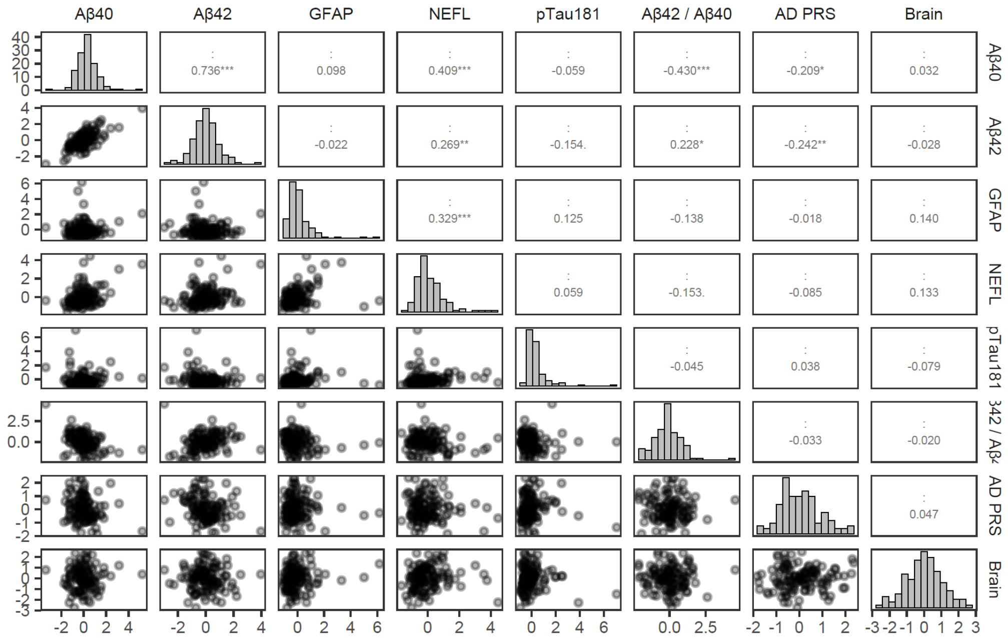

2. **Genetic determinants of organ aging clock**

After identifying the unique role of brain aging across neuropsychiatric disorders, and the role of systematic aging across multiple organs in physical diseases, we aimed to characterize the genetic determinants of brain aging and organismal aging. We conducted GWAS on brain and organismal age gap in 29,629 UKB participants of European ancestry.

For brain age gap, 38 independent genome-wide significant SNPs within 6 genomic risk loci were mapped to 149 protein-coding genes (**Extended Data Fig. 5**). The 5 top ranked genes included *GABBR1*,[17-20] *ECM1*,[21] *TARS2*,[22] *ARNT*,[23] and *CA14*,[24] which had known roles in neurodegenerative diseases and brain health. *GABBR1* gene encodes gamma-aminobutyric acid (GABA) receptors that involved in the GABAergic neurotransmission of the central nervous system. GABAergic dysregulation is considered as an early biomarker of AD-related cognitive decline and plays a central role in psychiatric and neurological disorders, as well as normal aging.[17-20] Notably, GABA receptors represent novel therapeutic targets at cognitive symptoms and neuropsychiatric disorders such as depression,[18,20] schizophrenia,[19] and AD[17]. *ECM1* that encodes extracellular matrix protein 1, as one component of extracellular matrix (ECM), was involved in the biological aging and age-related diseases such as heart failure.[21] *TARS2* gene was reported to be related to neurodevelopmental disorder and intellectual disability.[22] *ARNT* gene that encodes aryl hydrocarbon receptor (AhR) nuclear translocator protein that forms a complex with ligand-bound AhR for receptor functioning. AhR has been considered to be involved in aging phenotypes and the hallmarks of brain aging.[23] *CA14* was associated with processing speed and had causal effects on hippocampal volume.[24] Enrichment analyses of all mapped genes showed significant gene overlap with previously identified genes for psychiatric disorders and brain morphology in GWAS catalog. Tissue enrichment analysis showed no significant tissue overexpression of related genes. This highlights that the genes regulating the brain aging clock as proxied by brain-enriched proteins are generally expressed in multiple tissues. This finding aligns with a recent GWAS on phenotypic brain aging,[25] suggesting a potential systemic basis for the genetic architecture of brain aging.

For organismal age gap, 33 independent GWS SNPs within 8 genomic risk loci were mapped to 46 protein-coding genes (**Extended Data Fig. 6**). The 5 top ranked genes included *KLHL22*, *MED15, SCARF2*, *ZNF74*, and *SMAD5*, which had known roles in aging and age-related diseases. *KLHL22* was reported to promote ageing and tumorigenesis and may have therapeutic potential for treatment of age-related diseases.[26] *MED15* that encodes component of the mediator complex was related to low-temperature–induced longevity and organismal fitness in *C. elegans* via the maintenance of lipidostasis and proteostasis,[27] and to various human neurological disorders and cancer.[28] *SCARF2* was associated with COPD,[29] and *ZNF74* was related to age-at-onset of schizophrenia.[30] Tissue enrichment analysis showed no significant tissue overexpression of genes related to organismal aging.

Reference

17 Carello-Collar, G. *et al.* The GABAergic system in Alzheimer's disease: a systematic review with meta-analysis. *Molecular Psychiatry* **28**, 5025-5036, doi:10.1038/s41380-023-02140-w (2023).

18 Luscher, B., Maguire, J. L., Rudolph, U. & Sibille, E. GABA(A) receptors as targets for treating affective and cognitive symptoms of depression. *Trends Pharmacol Sci* **44**, 586-600, doi:10.1016/j.tips.2023.06.009 (2023).

19 Xu, M. Y. & Wong, A. H. C. GABAergic inhibitory neurons as therapeutic targets for cognitive impairment in schizophrenia. *Acta Pharmacol Sin* **39**, 733-753, doi:10.1038/aps.2017.172 (2018).

20 Cryan, J. F. & Kaupmann, K. Don't worry 'B' happy!: a role for GABAB receptors in anxiety and depression. *Trends in Pharmacological Sciences* **26**, 36-43, doi:https://doi.org/10.1016/j.tips.2004.11.004 (2005).

21 Guvatova, Z. G., Borisov, P. V., Alekseev, A. A. & Moskalev, A. A. Age-Related Changes in Extracellular Matrix. *Biochemistry (Mosc)* **87**, 1535-1551, doi:10.1134/s0006297922120112 (2022).

22 Accogli, A. *et al.* Clinical, neuroradiological, and molecular characterization of mitochondrial threonyl-tRNA-synthetase (TARS2)-related disorder. *Genet Med* **25**, 100938, doi:10.1016/j.gim.2023.100938 (2023).
23 Ojo, E. S. & Tischkau, S. A. The role of AhR in the hallmarks of brain aging: Friend and foe. *Cells* **10**, 2729 (2021).
24 Walker, R. M. *et al.* The circulating proteome and brain health: Mendelian randomisation and cross-sectional analyses. *Transl Psychiatry* **14**, 204, doi:10.1038/s41398-024-02915-x (2024).
25 Wen, J. *et al.* The genetic architecture of biological age in nine human organ systems. *Nature Aging*, doi:10.1038/s43587-024-00662-8 (2024).
26 Chen, J. *et al.* KLHL22 activates amino-acid-dependent mTORC1 signalling to promote tumorigenesis and ageing. *Nature* **557**, 585-589, doi:10.1038/s41586-018-0128-9 (2018).
27 Lee, D. *et al.* MDT-15/MED15 permits longevity at low temperature via enhancing lipidostasis and proteostasis. *PLoS Biol* **17**, e3000415, doi:10.1371/journal.pbio.3000415 (2019).
28 Allen, B. L. & Taatjes, D. J. The Mediator complex: a central integrator of transcription. *Nature reviews Molecular cell biology* **16**, 155-166 (2015).
29 Wang, S., Yue, Y., Wang, X., Tan, Y. & Zhang, Q. SCARF2 is a target for chronic obstructive pulmonary disease: Evidence from multi-omics research and cohort validation. *Aging Cell*, e14266, doi:10.1111/acel.14266 (2024).
30 Takase, K. *et al.* Association of ZNF74 gene genotypes with age-at-onset of schizophrenia. *Schizophrenia research* **52**, 161-165 (2001).

3. **Brain and body aging with brain structural changes**
   After identifying the molecular links between the brain aging clock and AD, we tested the associations of organ aging with structural brain changes during follow-up, as both biological aging and neurodegenerative diseases can lead to progressive cerebral atrophy and changes in brain structures (**Extended Data Fig. 7**). Overall, both brain and organismal aging were associated with the reduced total brain volume, total GMV, and increased tWMH. Organ aging was significantly related to reduced cortical grey matter volumes, especially in orbitofrontal cortex, precentral gyrus, superior frontal gyrus, paracingulate gyrus, insular cortex, and superior and middle temporal gyrus. Organ aging, especially in the digestive and endocrine organs, were also related to reduced GMV in multiple subcortical and cerebellar regions. We then assessed the association of brain and organismal aging with white matter microstructure indices across the white matter tract regions. Generally, brain aging was negatively related to regional FA, ICVF, and OD, but positively to MD and ISOVF. The white matter tracts mainly involved included CT, FMA, IFO, ILF, PTR, SLF, and STR. Organismal aging was negatively related to OD but positively to MD and ISOVF.

4. **Brain and body aging in mental well-being and psychiatric diseases and featured pathways**
   We included age gap in brain, intestine, pancreas and kidney that independently associated with the increased risk of psychiatric diseases. Aging in these four organs was related to the baseline PHQ-4 scores and depressive and anxiety symptoms (**Extended Data Fig. 8a**). Intestine and kidney aging were also related to future mental health conditions, such as PHQ-9 score and depressive symptoms, in healthy participants (**Extended Data Fig. 8b**). Intestine and brain aging were associated with increased risk of depression, SUD, and mortality in participants with psychological distress at baseline (**Extended Data Fig. 8c**), which was similar to the trends of association in the full sample (**Extended Data Fig. 8d**). Brain age gap could predict future depression, independent of PHQ-4 and PRS (**Extended Data Fig. 8e**), and the combination of brain aging and PRS stratified the risk of incident depression (**Extended Data Fig. 8f**).

   The aging trajectories of prioritized proteins related to both organ aging and depression/anxiety suggested that brain proteins changed earlier and faster with age than proteins of other digestive/endocrine organs (**Extended Data Fig. 8g-h**). The top prioritized proteins were specifically expressed in brain or intestine (**Extended Data Fig. 8i**). We identified a protein interaction network using StringDB (**Extended Data Fig. 8i**), including proteins in brain aging clock (NEFL, RBFOX3, MOG, CNTN2) and connected proteins (e.g., MAG, MBP, CNP, CNTNAP1). NEFL, as a validated biomarkers for neurological conditions, was also related to multiple psychiatric disorders and symptom severity.[53] *RBFOX3* that regulates neurogenesis and synaptogenesis have been implicated in GWAS of major depression[54] and sleep latency[55]. Notably, the network included a number of proteins encoded by oligodendrocyte (OL) lineage-related genes that are important for myelination and myelin structure and have been implicated in psychiatric disorders, including myelin oligodendrocyte glycoprotein (MOG), CNP, MAG, and MBP.[56] For instance, *CNP* dysfunction can induce subtle white matter abnormalities with neurodegenerative changes and cause/amplify multiple neuropsychiatric disorders, especially upon aging.[57] Another protein network of SPINK family, closely related to pancreatitis,[58] was identified, showing biological enrichment in the negative regulation of proteolysis (**Extended Data Fig. 8i-j**). Depression and anxiety are common psychiatric comorbidities in patients with chronic pancreatitis.[59] These findings are align with the diverse mechanisms in the pathogenesis of major depression, such as neurogenesis and neuroplasticity hypotheses, and the involvement of OL lineage cells that supports myelin formation and synaptic transmission.[56,60]

   Despite a shared genetic and molecular basis across major psychiatric and neurodegenerative diseases, the pathogenic pathways linked by the organ aging clocks to psychiatric diseases are largely distinct from those associated with dementia, suggesting the potential specificity of the organ aging clock in predicting diseases with shared mechanisms.

Reference

53 Bavato, F. *et al.* Introducing neurofilament light chain measure in psychiatry: current evidence, opportunities, and pitfalls. *Molecular Psychiatry*, doi:10.1038/s41380-024-02524-6 (2024).

54 Wray, N. R. *et al.* Genome-wide association analyses identify 44 risk variants and refine the genetic architecture of major depression. *Nat Genet* **50**, 668-681, doi:10.1038/s41588-018-0090-3 (2018).
55 Amin, N. *et al.* Genetic variants in RBFOX3 are associated with sleep latency. *Eur J Hum Genet* **24**, 1488-1495, doi:10.1038/ejhg.2016.31 (2016).
56 Zhou, B., Zhu, Z., Ransom, B. R. & Tong, X. Oligodendrocyte lineage cells and depression. *Molecular Psychiatry* **26**, 103-117, doi:10.1038/s41380-020-00930-0 (2021).
57 Hagemeyer, N. *et al.* A myelin gene causative of a catatonia-depression syndrome upon aging. *EMBO Mol Med* **4**, 528-539, doi:10.1002/emmm.201200230 (2012).
58 Pfützer, R. H. *et al.* SPINK1/PSTI polymorphisms act as disease modifiers in familial and idiopathic chronic pancreatitis. *Gastroenterology* **119**, 615-623, doi:10.1053/gast.2000.18017 (2000).
59 Phillips, A. E. *et al.* Psychiatric Comorbidity in Patients With Chronic Pancreatitis Associates With Pain and Reduced Quality of Life. *Am J Gastroenterol* **115**, 2077-2085, doi:10.14309/ajg.0000000000000782 (2020).
60 Cui, L. *et al.* Major depressive disorder: hypothesis, mechanism, prevention and treatment. *Signal Transduction and Targeted Therapy* **9**, 30, doi:10.1038/s41392-024-01738-y (2024).

5. Proteomic profiling in the CKB

We included CKB participants with baseline (2004-2008) OLINK data in a nested case-cohort study of IHD, and who were not genetically related (n = 3,977), including 1951 incident IHD cases and a randomly selected sub-cohort of 2026 participants. The participants had no prior history of CVD, no history of lipid-lowering drug (e.g., statins) use at the blood sample collection. Baseline plasma samples from participants were retrieved, thawed, and aliquoted into 96-well plates, with 8 wells reserved for quality control (QC) assays. Each plate contained a mixture of cases and controls, arranged in the order they were retrieved from storage at the Wolfson laboratory in Oxford. To minimize variation within and between runs, the samples were randomly distributed across plates and normalized using both an internal control and an inter-plate control. A pre-determined correction factor was then applied to the data. Plasma levels of 2,923 proteins were measured using the OLINK Explore 3072 panel, processed in two separate batches. The first batch (1,463 proteins) was analyzed at the OLINK laboratory in Uppsala, Sweden, while the second batch (1,460 proteins) was processed at the OLINK laboratory in Boston, USA, with each batch covering all samples. The limit of detection (LOD) was established using negative control samples (buffer without antigen). Samples were flagged with a QC warning if the incubation control deviated by more than a pre-set value (±0.3) from the median of all plate samples, though values below the LOD were still included in the analysis. Individual samples were flagged for assay warnings if values deviated by 3-fold or more from negative controls. The processed data were provided in arbitrary normalized protein eXpression units on a log2 scale.

6. Proteomic profiling in the NHS

We included NHS participants with OLINK data in a nested case-cohort study of colon cancer within the NHS blood sub-cohort (n = 800) with blood samples collected between 1989 and 1990. In NHS (mean age = 57 years), participants received a mailed blood collection kit containing a consent form, supplies, and instructions for venipuncture. Samples were returned via prepaid overnight courier in a Styrofoam container with an ice pack. Blood was drawn into heparin tubes, centrifuged upon arrival, and separated into buffy coat, red blood cells, and plasma. Aliquots were stored in liquid nitrogen freezers at ≤ −130°C, with processing completed within hours of receipt; most samples (>90%) were frozen within 30–36 hours of collection. Proteomic profiling was conducted using OLINK's immunoaffinity-based platform (Uppsala, Sweden), covering four panels (cardiometabolic, inflammation, neurology, and oncology) that measure 2,923 independent proteins. The OLINK technology employs a proximity extension assay (PEA), a highly multiplexed immune-PCR method that minimizes cross-reactivity. PEA utilizes two nucleotide-labeled antibodies targeting non-overlapping epitopes of each analyte. Upon binding, the oligonucleotides hybridize, undergo DNA polymerase-mediated extension, and generate a PCR amplicon, which is subsequently quantified via microfluidic qPCR. OLINK data were normalized and transformed using internal and interplate controls to correct for intra- and inter-run variability. The final assay read-out is in Normalized Protein eXpression (NPX), which is an arbitrary unit on a log-scale, where a higher value corresponds to a higher protein expression. A predefined lower limit of detection (LOD) based on negative controls were included in each run, with values below the LOD set to missing.

7. Model benchmarking

We refer to the results from Argentieri et al. for the performance of various machine-learning models (LASSO, elastic net, LightGBM, ResNet, MLP, TabR) using plasma proteomics to predict organismal age. Models were trained on the UKB training set and tested on the UKB holdout test set (with a sample size similar to the organismal aging clock training in our study), as well as validation sets from the CKB and FinnGen cohorts (*Argentieri, M.A., Xiao, S., Bennett, D. et al. Proteomic aging clock predicts mortality and risk of common age-related diseases in diverse populations. Nat Med 30, 2450–2460 (2024). https://doi.org/10.1038/s41591-024-03164-7*).

Model performance for estimation of proteomic organismal age across cohorts

	UKB	CKB	FinnGen
LASSO	0.9329	0.9167	0.9336
Elastic Net	0.9331	0.9175	0.9341
LightGBM	0.9373	**0.9217**	**0.9368**
ResNet	**0.9384**	0.9145	0.9224
MLP	0.9362	0.9043	0.8960
TabR	0.9373	0.8969	0.8615

LightGBM achieved the second-best model accuracy in the UKB test set but demonstrated significantly better performance in the independent validation sets.

8. Calculation of KDM-BA and PhenoAge

PhenoAge was calculated based on albumin, alkaline phosphatase, C-reactive protein, glucose, lymphocyte proportion, mean cell volume, white blood cell count, red cell distribution width. KDM-BA was calculated based on albumin, alkaline phosphatase, C-reactive protein, creatinine, HbA1c, total cholesterol, blood urea nitrogen, and systolic blood pressure. Computation of PhenoAge and KDM-BA was conducted using the R package 'BioAge' (https://github.com/dayoonkwon/BioAge). Briefly, biological aging algorithms were trained in NHANES III and project biological aging measures in the UKB.

NHANES is an ongoing nationally representative, cross-sectional survey conducted by the US Centers for Disease Control and Prevention. NHANES administers questionnaires during in-home interviews and conducts health examinations, including blood draws, in a mobile examination center. Details of recruitment procedures and study design are available from the Center for Disease Control and Prevention (https://www.cdc.gov/nchs/nhanes/index.htm).

PhenoAge

The PhenoAge algorithm is derived from multivariate analysis of mortality hazards. Initially, it was constructed using elastic-net Gompertz regression to predict mortality from 42 biomarkers in the NHANES III dataset. This current algorithm identified nine biomarkers and chronological age as key predictors, forming a parsimonious model. The model is used to compute a mortality prediction score. An individual's PhenoAge prediction corresponds to the chronological age at which their mortality risk would be approximately normal in a reference population. The formula is:

$$\text{PhenoAge} = 141.50225 + \frac{\ln[-0.00553 \times \ln(1-\text{mortality risk})]}{0.090165}$$

where

$$\text{mortality risk} = 1 - e^{-e^{xb}[\exp(120_{x\gamma})-1]/\gamma}$$

$$\gamma = 0.0076927$$

$$\begin{aligned} xb = & -19.907 - 0.0336 + \text{albumin} + 0.0095 \times \text{creatinine} + 0.1953 \\ & \times \text{glucose} + 0.0954 \times \ln(C - \text{reactive protein}) - 0.012 \\ & \times \text{lymphocyte percentage} + 0.0268 \times \text{mean corpuscular volume} \\ & + 0.3306 \times \text{red cell distribution width} + 0.00188 \times \text{alkaline phosphatase} \\ & + 0.0554 \times \text{white blood cell count} + 0.0804 \times \text{chronological age} \end{aligned}$$

KDM-BA

An individual's KDM BA prediction corresponds to the chronological age at which her/his physiology would be approximately normal. The KDM BA algorithm is derived from a series of regressions of individual biomarkers on chronological age in a reference population. The equation takes information from *n* number of regression lines of chronological age regressed on *n* biomarkers.

$$BA_{\mathrm{EC}} = \frac{\sum_{\mathrm{i}=1}^{\mathrm{n}} (x_{\mathrm{i}} - q_{\mathrm{i}}) \frac{k_{\mathrm{i}}}{s_{\mathrm{i}}^2} + \frac{CA}{s_{\mathrm{BA}}^2}}{\sum_{\mathrm{i}=1}^{\mathrm{n}} \left(\frac{k_{\mathrm{i}}}{s_{\mathrm{i}}}\right)^2 + \frac{1}{s_{\mathrm{BA}}^2}}$$

where x is the value of biomarker i measured for an individual. For each biomarker i, the parameters k, q, and s are estimated from a regression of chronological age on the biomarker in the reference sample. k, q, and s are the regression intercept, slope, and root mean squared error, respectively. sBA is a scaling factor equal to the square root of the variance in chronological age explained by the biomarker set in the reference sample. CA is chronological age. In the BioAge package, the reference sample is NHANES III nonpregnant participants aged 30–75 years. Algorithm parameters are estimated separately for men and women.

Supplementary Figures

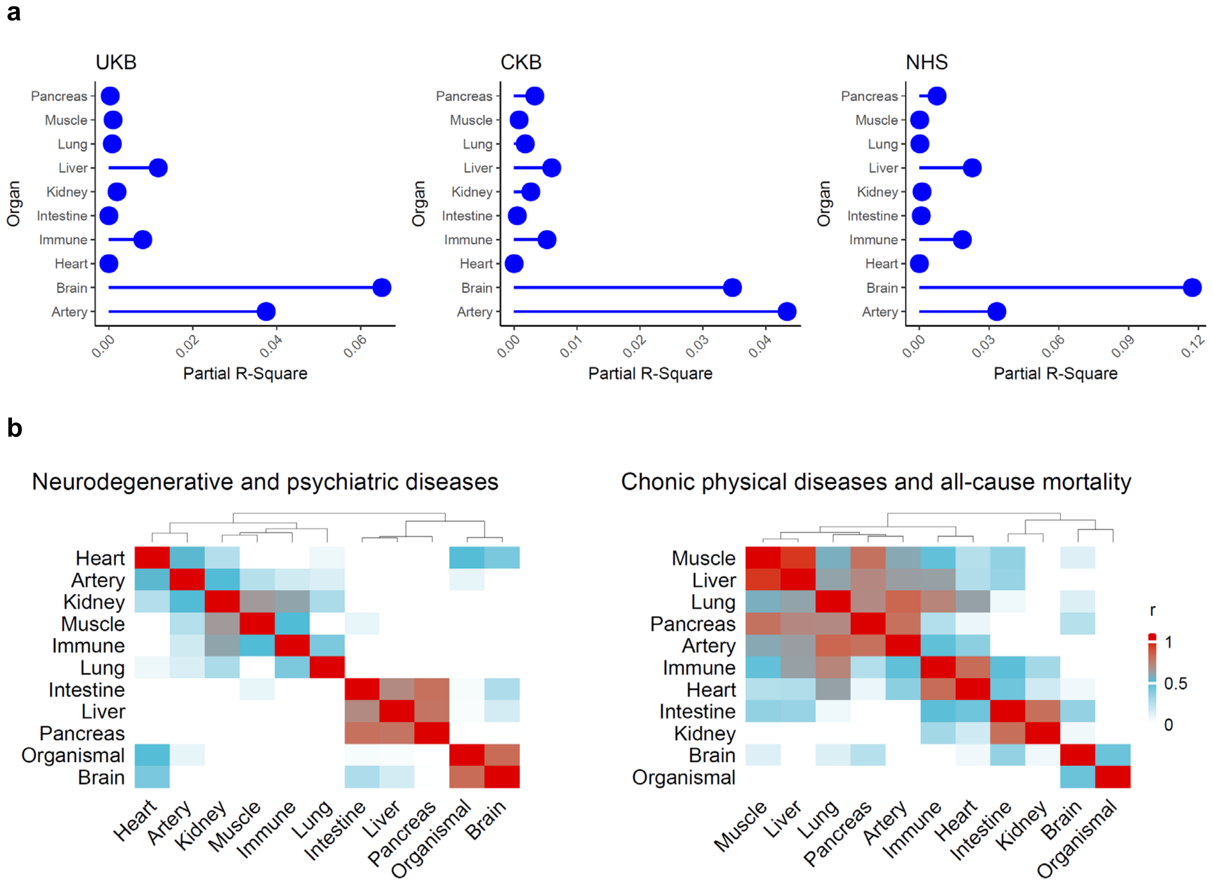

Supplementary Figure 1. Correlation patterns of organ aging clock. **a**, Partial R^2 for each organ age: a linear model was fitted with multiple organ proteomic ages as predictors of chronological age. **b**, correlation of association Z-scores for different outcome phenotypes (with all clocks included in the multivariable Cox model), categorized into neuropsychiatric diseases and chronic physical diseases, across organ aging clocks in the UKB.

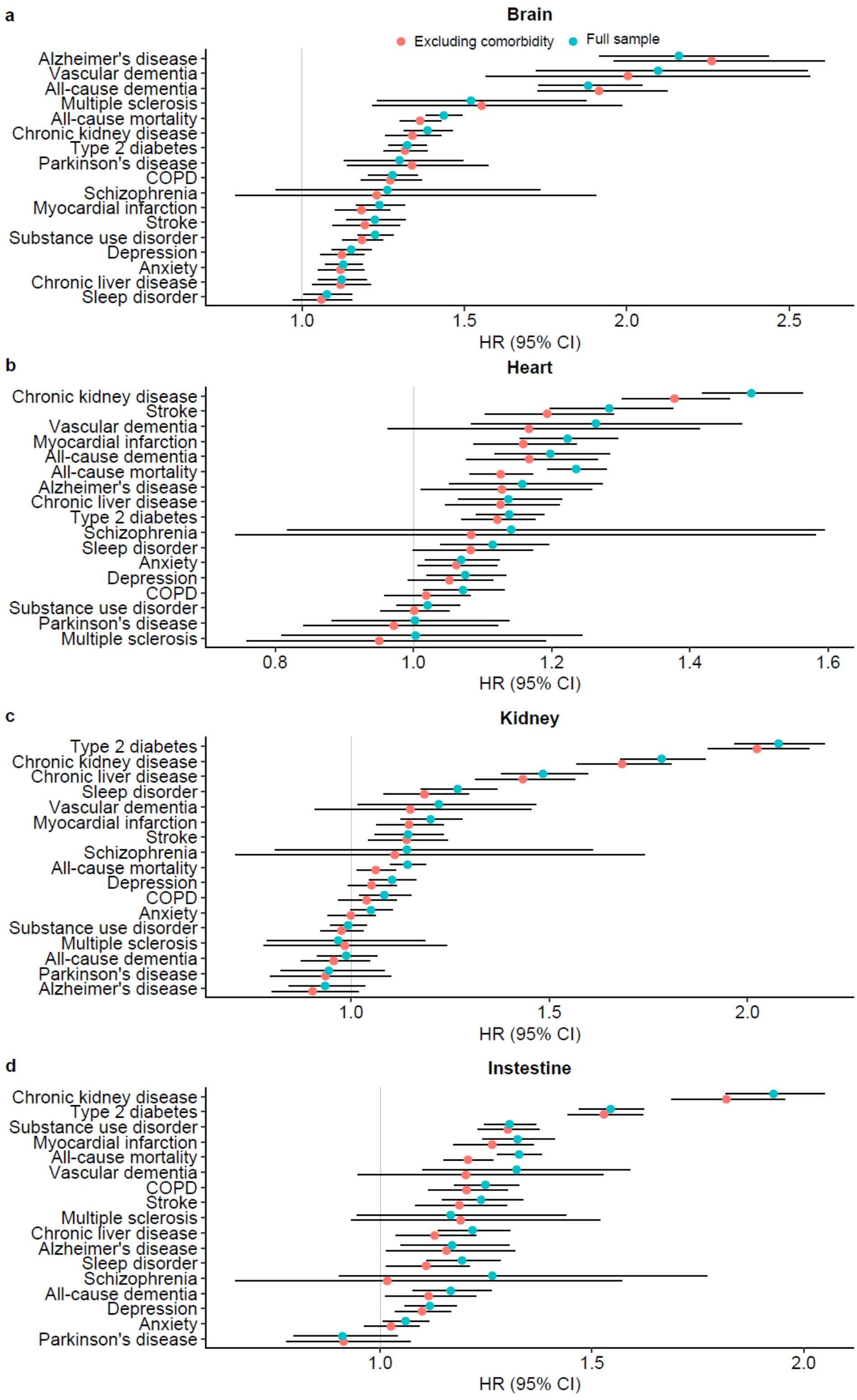

Supplementary Figure 2. Association of proteomic aging clock and association with diseases and mortality after accounting for baseline comorbidities related to organ-specific aging clock. The associations between baseline comorbidities (time since prevalent disease) and organ aging clock are shown in **Fig.1g**. **a,** The association of *brain* aging clock with diseases and mortality in full UKB sample (*n* = 43,616) versus subsample excluding those with baseline comorbidities related to brain aging clock (*n* = 35,057). Prevalent comorbidities related to brain aging clock include multiple sclerosis, Parkinson's disease, depression, substance use disorders, schizophrenia, COPD, stroke, type 2 diabetes, and chronic kidney disease. **b,** The association of *heart* aging clock with diseases and mortality in full sample (*n* = 43,616) versus subsample excluding those with baseline

comorbidities related to heart aging clock (*n* = 39,446). Related prevalent comorbidities excluded include Parkinson's disease, myocardial infarction, COPD, stroke, type 2 diabetes, and chronic kidney disease. **c,** The association of *kidney* aging clock with diseases and mortality in full sample (*n* = 43,616) versus subsample excluding those with baseline comorbidities related to heart aging clock (*n* = 35,624). Related prevalent comorbidities excluded include depression, sleep disorder, myocardial infarction, schizophrenia, COPD, stroke, type 2 diabetes, and chronic kidney disease. **d,** The association of *intestine* aging clock with diseases and mortality in full sample (*n* = 43,616) versus subsample excluding those with baseline comorbidities related to intestine aging clock (*n* = 39,446). Related prevalent comorbidities excluded include depression, substance use disorders, myocardial infarction, schizophrenia, COPD, stroke, type 2 diabetes, and chronic kidney disease. All Cox regression models were adjusted for age, sex, ethnicity, Townsend deprivation index, physical activity level, and recruitment center.

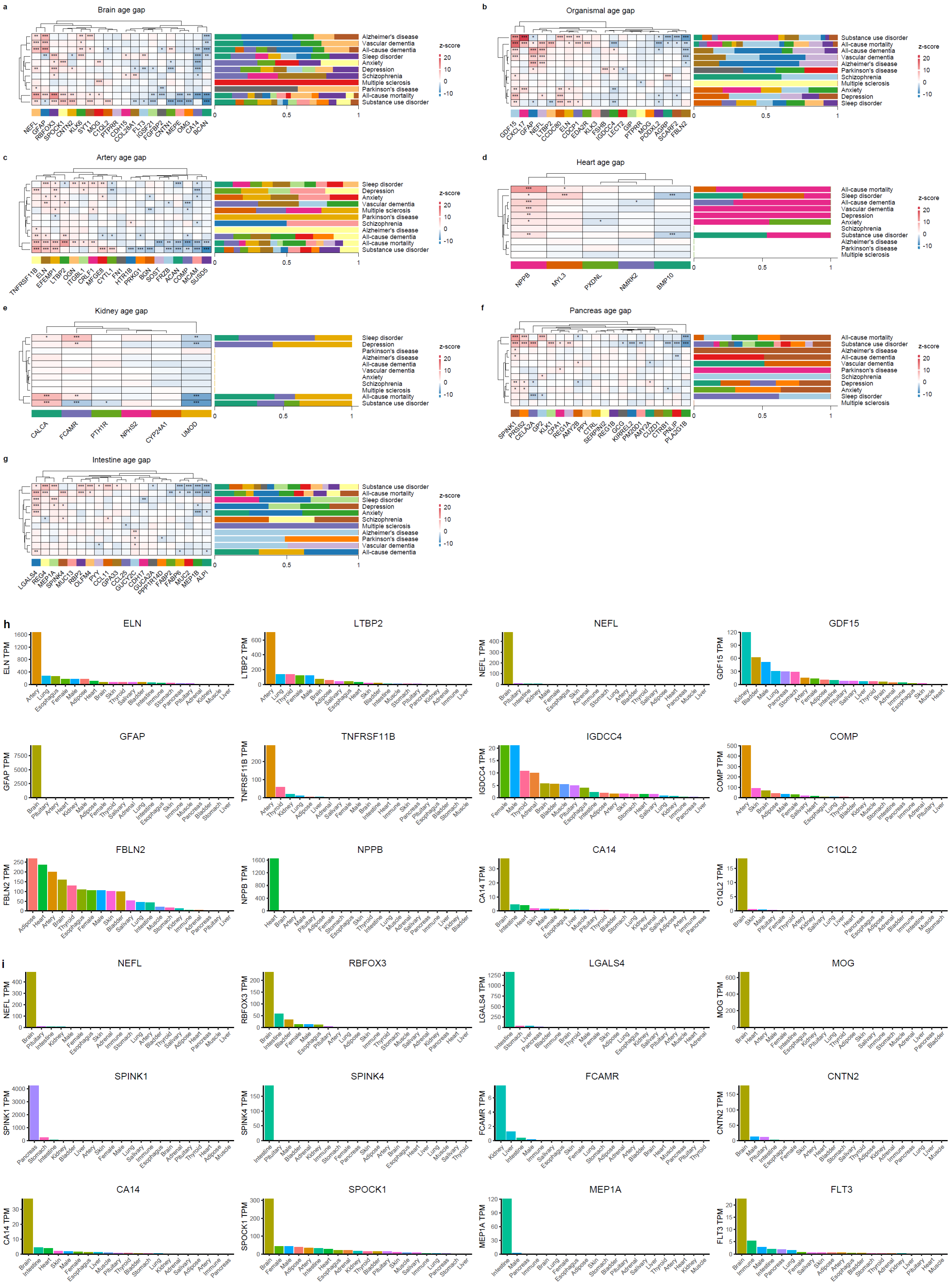

Supplementary Figure 3. Importance of top proteins in each organ-specific aging clock for neuropsychiatric diseases and the expression of prioritized proteins associated with both organ aging and neuropsychiatric diseases. a-g, Protein importance for each disease studied. For each outcome, Cox model was calculated using all the top 20 proteins of brain aging, with adjustment for age, sex, ethnicity, Townsend deprivation index, physical activity level, and recruitment center. In the left panel, the association of individual protein and incident outcome is colored by z-score, with z-scores for insignificant

associations (P value ≥ 0.05) set as 0. In the right panel, relative importance of proteins significantly associated with outcomes is shown. Relative importance was calculated for each outcome by scaling z-score for significant association so that their sum equals 1. Only organ aging clocks independently associated with the risk of psychiatric (kidney, brain, intestine, pancreas) and neurodegenerative (brain, heart, organismal, and artery) diseases were shown. **h,** Prioritized proteins associated with both aging and NDs. **i**, Prioritized proteins associated with both aging and psychiatric diseases. Expression levels of prioritized proteins across tissues in GTEx were illustrated.

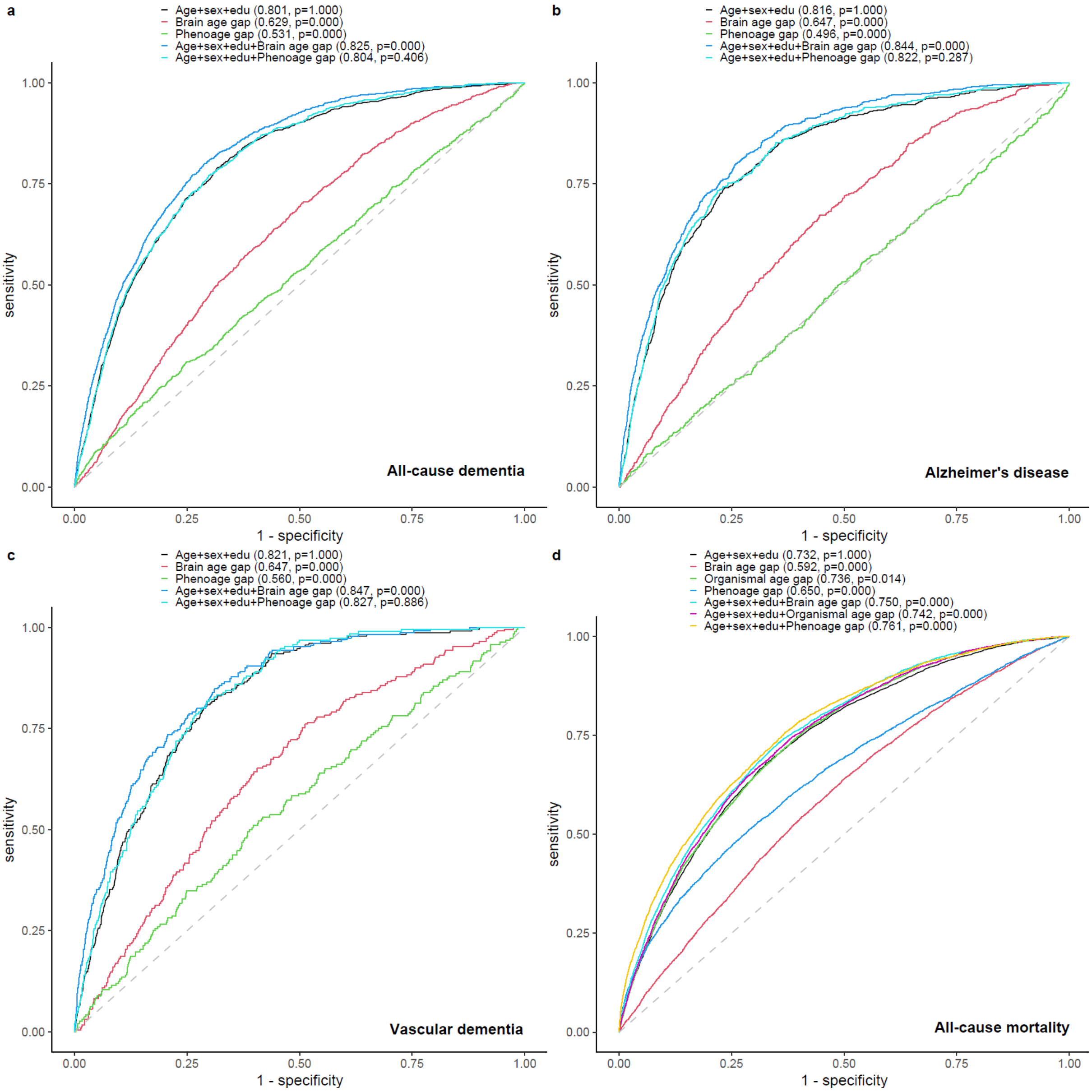

Supplementary Figure 4. Comparison of predictive performance between proteomic aging clock (brain and organismal) and basic demographic factors (age, sex, and education) for dementia and mortality. Receiver operating curves of the predictive performance of proteomic aging clock versus basic demographic factors (age, sex, and education) for all cases of incident all-cause dementia (**a**), Alzheimer's disease (**b**), vascular dementia (**c**), and all-cause mortality (**d**).

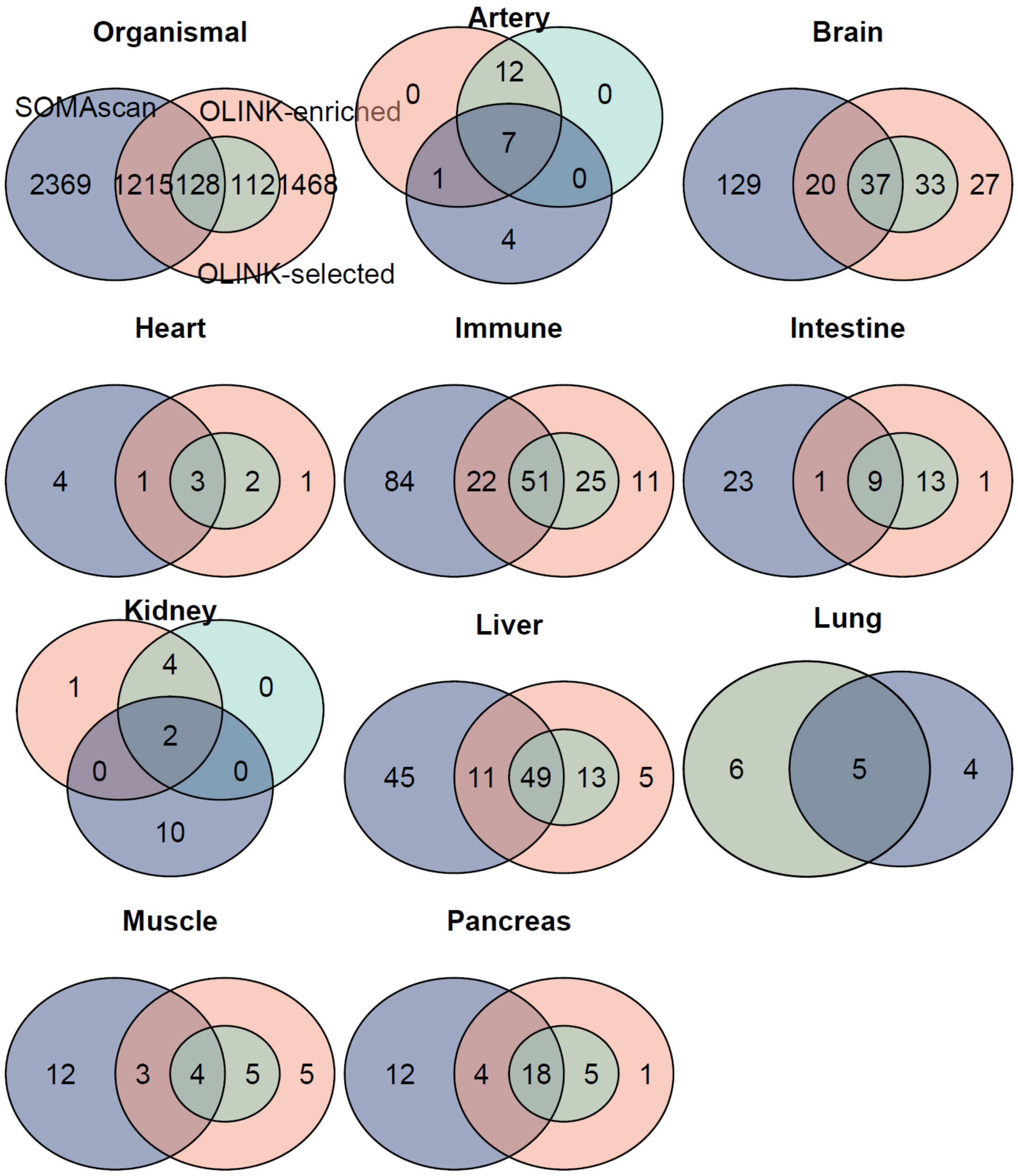

Supplementary Figure 5. Overlap in numbers of protein signatures between the OLINK 3072-based organ aging clock and the SOMAscan-based clock (Oh et al., 2023).

Supplementary table descriptions.

Supplementary Table 1. Characteristics of study participants in the UKB, CKB and NHS.

Supplementary Table 2. Tissue to organ mapping in GTEx (v8). Organ-enriched genes and plasma proteins were determined using the human organ bulk RNA sequencing (RNA-seq) data from the Genotype-Tissue Expression (GTEx) project (v8, 54 tissue types). Tissues in GTEx were mapped to corresponding organs according to the physiological function. Organ-level gene expression was established by identifying the maximum expression value among its tissue subtypes.

Supplementary Table 3. Details of proteins on the Olink Explore 3072 platform with annotation of organ enrichment. Genes were defined as organ-enriched when their expression exhibited a fourfold increase in one organ compared to any others, in accordance with the criteria by the HPA that was used and validated in previous studies. 418 proteins out of 2,916 Olink proteins (14.3%) were identified as enriched in at least one of ten major organs or systems, including brain, heart, lung, immune system, artery, intestine, liver, kidney, muscle, and pancreas.

Supplementary Table 4. Age-related proteins in organ aging clocks. Light gradient boosting machine (LightGBM) model was used to identify age-related proteins.

Supplementary Table 5. Correlation of proteomic organ age with chronological age across cohorts. Performance was assessed by Pearson correlations between predicted organ age and chronological age in the UKB, CKB and NHS.

Supplementary Table 6. Correlation patterns of organ age gap across cohorts. Pairwise correlations among organismal and organ-specific age gaps in the UKB, CKB, and NHS.

Supplementary Table 7. Association of organ aging with incident diseases and death in the UKB. Summary statistics from Cox proportional hazards models between organ age gaps and incident diseases and death in the UKB, with adjustment for age, sex, ethnicity, Townsend deprivation index, smoking, physical activity level, and recruitment center.

Supplementary Table 8. Association of organ aging with incident diseases and death in the CKB. Summary statistics from Cox proportional hazards models between organ age gaps and incident diseases and death in the CKB, with adjustment for age, sex, ethnicity, education, study region, smoking, and physical activity level.

Supplementary Table 9. Association of organ aging with incident diseases and death in the NHS. Summary statistics from Cox proportional hazards models between organ age gaps and incident diseases and death in the NHS, with adjustment for age, ethnicity, neighborhood socioeconomic status, smoking, and physical activity level.

Supplementary Table 10. Correlation of disease progression with organ aging. Summary statistics from linear regressions between organ age gaps and years since disease diagnosis in participants with prevalent diseases at baseline proteomic assessment.

Supplementary Table 11. Association of extreme ageotypes with incident diseases and death in the UKB. Summary statistics from Cox proportional hazards models between organ ageotypes and incident diseases and death in the UKB, with adjustment for age, sex, ethnicity, Townsend deprivation index, smoking, physical activity level, and recruitment center.

Supplementary Table 12. Definition of disease outcomes in the UKB.

Supplementary Table 13. Definition of age-related phenotypes and covariables in the UKB.

Supplementary Table 14. Definition of disease outcomes in the CKB.

Supplementary Table 15. Independent genome-wide significant SNPs for brain aging.

Supplementary Table 16. Overlap and enrichment of genes of brain aging in GWAS catalog reported gene set.

Supplementary Table 17. Independent genome-wide significant SNPs for organismal aging.

Supplementary Table 18. Overlap and enrichment of genes of organismal aging in GWAS catalog reported gene set.
