# Supplementary material for: Organ-specific proteomic aging clocks predict disease and longevity across diverse populations
Source: Nat Aging. 2025 Nov 26;6(1):162–80. doi: 10.1038/s43587-025-01016-8 (PMC12823432; doi:10.1038/s43587-025-01016-8)
Supplement: Supplementary file 2 — Reporting Summary [file 43587_2025_1016_MOESM2_ESM.pdf]

nature portfolio

Corresponding author(s): Yunhe Wang

Last updated by author(s): 9/15/2025

Reporting Summary

Nature Portfolio wishes to improve the reproducibility of the work that we publish. This form provides structure for consistency and transparency in reporting. For further information on Nature Portfolio policies, see our Editorial Policies and the Editorial Policy Checklist.

Statistics

For all statistical analyses, confirm that the following items are present in the figure legend, table legend, main text, or Methods section.

n/a	Confirmed	
☐	☒	The exact sample size (*n*) for each experimental group/condition, given as a discrete number and unit of measurement
☐	☒	A statement on whether measurements were taken from distinct samples or whether the same sample was measured repeatedly
☐	☒	The statistical test(s) used AND whether they are one- or two-sided<br>*Only common tests should be described solely by name; describe more complex techniques in the Methods section.*
☐	☒	A description of all covariates tested
☐	☒	A description of any assumptions or corrections, such as tests of normality and adjustment for multiple comparisons
☐	☒	A full description of the statistical parameters including central tendency (e.g. means) or other basic estimates (e.g. regression coefficient) AND variation (e.g. standard deviation) or associated estimates of uncertainty (e.g. confidence intervals)
☐	☒	For null hypothesis testing, the test statistic (e.g. *F*, *t*, *r*) with confidence intervals, effect sizes, degrees of freedom and *P* value noted<br>*Give P values as exact values whenever suitable.*
☒	☐	For Bayesian analysis, information on the choice of priors and Markov chain Monte Carlo settings
☐	☒	For hierarchical and complex designs, identification of the appropriate level for tests and full reporting of outcomes
☐	☒	Estimates of effect sizes (e.g. Cohen's *d*, Pearson's *r*), indicating how they were calculated

Our web collection on statistics for biologists contains articles on many of the points above.

Software and code

Policy information about availability of computer code

Data collection	No software was used (data used is directly available from UK Biobank (UKB), China Kadoorie Biobank (CKB), and Nurses' Health Study (NHS) as detailed in the methods).
Data analysis	All analyses and data visualizations were conducted using R (v.4.2) and Python (v.3.6). REGENIE was used to conduct genome-wide association analysis on the UKB RAP.

For manuscripts utilizing custom algorithms or software that are central to the research but not yet described in published literature, software must be made available to editors and reviewers. We strongly encourage code deposition in a community repository (e.g. GitHub). See the Nature Portfolio guidelines for submitting code & software for further information.

Data

Policy information about availability of data

All manuscripts must include a data availability statement. This statement should provide the following information, where applicable:

- Accession codes, unique identifiers, or web links for publicly available datasets
- A description of any restrictions on data availability
- For clinical datasets or third party data, please ensure that the statement adheres to our policy

Researchers can apply to use the UK Biobank dataset by registering and applying at https://ukbiobank.ac.uk/register-apply/. Use of UK Biobank was approved by UK Biobank Ethics Advisory Committee under application 98358. The CKB is a global resource for the investigation of lifestyle, environmental, blood biochemical and

genetic factors as determinants of common diseases. The CKB study group is committed to making the cohort data available to the scientific community in China, the United Kingdom and worldwide to advance knowledge about the causes, prevention and treatment of disease. For detailed information on what data are currently available to open access users, how to apply for them and the timeline for data access, please visit the CKB website: https://www.ckbiobank.org/data-access. Researchers who are interested in obtaining the raw data that have been officially released from the CKB study should contact ckbaccess@ndph.ox.ac.uk. A research proposal will be requested to ensure that any analysis is performed by bona fide researchers. For any data that are not currently available via open access, researchers may need to develop a formal collaboration with the CKB study group. Because of participant confidentiality and privacy concerns, NHS data are available upon written request. According to standard controlled access procedure, applications to use NHS resources will be reviewed by our External Collaborators Committee for scientific aims, evaluation of the fit of the data for the proposed methodology, and verification that the proposed use meets the guidelines of the Ethics and Governance Framework and the consent that was provided by the participants. Investigators wishing to use NHS data are asked to submit a brief description of the proposed project (contact: nhsaccess@channing.harvard.edu). Investigators can expect initial responses within 4 weeks of request submission, as detailed on https://www.nurseshealthstudy.org/researchers. Human organ bulk RNA sequencing data from the Genotype-Tissue Expression project and Human Protein Atlas are available at https://www.gtexportal.org/home/downloads/adult-gtex/bulk_tissue_expression and https://www.proteinatlas.org/about/download, respectively. Preprocessed human single-cell RNA sequencing (scRNA-seq) data for brain,42 vasculature,48 were accessed from studies in the Human Cell Atlas (https://cellxgene.cziscience.com/gene-expression). Preprocessed human scRNA-seq data for brain vasculature were accessed from ref.47. Differential expression data of RNA and proteins between AD and control cases were assessed from ref.46. Any additional summary data generated and/or analyzed in the current study are available from the corresponding author on reasonable request.

Research involving human participants, their data, or biological material

Policy information about studies with human participants or human data. See also policy information about sex, gender (identity/presentation), and sexual orientation and race, ethnicity and racism.

Reporting on sex and gender

Our study included 43,616 participants from UKB (54% female, baseline age range: 37-70 years) and two independent external validation cohorts: 3,977 Chinese participants from the CKB (54% female, aged 30–78 years), and 800 US participants from the NHS (100% female, aged 43–69 years) (Supplementary Table 1). Plasma proteomic profiling was conducted in all three cohorts using the Olink Explore 3072 panel, measuring 2,916 proteins. Sex was further accounted for in the modeling of organ aging. As modeling of organismal aging suggested that sex-specific models were highly correlated with overall model for both sexes (r=0.99 and 0.98, respectively), we constructed models including both males and females to extend the generalizability of the findings. Sex was also used as a covariable throughout all association tests.

Reporting on race, ethnicity, or other socially relevant groupings

Of the 43,616 eligible participants with proteomic data in the UKB (54% female, mean age 57.4 (8.2) years, range 37-70 years), 93.1% are white, 2.4% black, 2.2% Asian, 0.6% mixed, 1.2% other ethnicity. Of the 3,977 eligible participants in the CKB (54% female, aged 30–78 years) with proteomic data, nearly 100% are Asian. Of the 800 eligible participants in the NHS (100% female, aged 43–69 years) with proteomic data, nearly 100% are white.

Population characteristics

Characteristics of the 43,616 eligible participants in the UKB for main analyses were as follows: 54% female, mean age 57.4 (8.2) years, range 37-70 years, 93.1% White, 10.7% current smoker, and 20.3% daily or almost daily drinker. Characteristics of the all 502,240 participants from the UKB cohort were as follows: 54% female; mean age 57.1 (8.1) years, age ranges: 37-70 years, 94.1% white, 10.5% current smoker, and 20.3% daily or almost daily drinker. The randomly selected participants with Olink proteomic data are representative of the general UKB population. A complete description of the demographic, lifestyle, and comorbid data of eligible participants are provided in the supplementary tables.

Recruitment

The UKB is a prospective population-based cohort of over 500,000 individuals aged 40-70 years who were recruited between 2006 to 2010 from the general population of the United Kingdom, with deep phenotyping and genomic data available.67 Participants were mainly followed up by data linkage to the electronic health and medical records, including national primary and secondary care, disease and mortality registries,68 with validated reliability, accuracy and completeness.69 Additional online surveys were conducted to enable the follow-up of cognitive and symptom-based mental well-being outcomes. In the current study, we included a subset of randomly selected, representative UKB participants with Olink proteomics data available at baseline (n =46,785).

The CKB is a prospective cohort study of 512,724 adults aged 30–79 years recruited from ten geographically diverse (five rural and five urban) areas across China during 2004 to 2008.70 We included CKB participants with baseline Olink data in a nested case–cohort study of IHD, and who were not genetically related (n = 3,977).

The NHS is a prospective cohort study of 121,700 female registered nurses in 11 US states, aged 30-55 years at enrollment in 1976, with follow-up data collected by biennial questionnaires.71 Between 1989 and 1990, 32,825 participants provided blood samples between 1989 and 1990. We included NHS participants with Olink data in a prospectively designed nested case–cohort study of colon cancer within the NHS (n = 800).

Ethics oversight

All contributing cohorts (UKB, CKB and NHS) received ethical approval from their respective institutional review boards and all participants consented to the use of their anonymized information for research purposes at the time of recruitment.

Note that full information on the approval of the study protocol must also be provided in the manuscript.

Field-specific reporting

Please select the one below that is the best fit for your research. If you are not sure, read the appropriate sections before making your selection.

☒ Life sciences ☐ Behavioural & social sciences ☐ Ecological, evolutionary & environmental sciences

For a reference copy of the document with all sections, see nature.com/documents/nr-reporting-summary-flat.pdf

Life sciences study design

All studies must disclose on these points even when the disclosure is negative.

Sample size	The UK Biobank (UKB) is a prospective population-based cohort of over 500,000 individuals aged 40-70 years recruited between 2006 and 2010, with detailed phenotyping and genomic data available. Of these, 54,219 participants underwent proteomic profiling of a baseline plasma sample using the OLINK Explore Assay with four panels (cardiometabolic, inflammation, neurology and oncology) measuring 2,941 independent proteins. The majority of participants (n=46,673) were randomly selected and the remainder were manually selected with enrichment for diseases of interest. We used the randomised subset, which was tested to be highly representative of the wider UKB population, to reduce selection bias. We further excluded participants with >20% missing proteins and seven proteins that were missing in over 20% of participants (GLIPR1, NPM1, PCOLCE, CST1, CTSS, TACSTD2 and ENDOU). The final discovery dataset included 43,616 participants with 2,916 proteins. To our knowledge, the UKB OLINK proteomic dataset is one of the largest to date, with detailed confounding and disease outcome information that was largely unavailable in previous studies. The large sample size obtained were deemed to provide robust modeling of proteomic biological aging and reliable risk estimates of health outcomes. We also included two validation cohorts including 3,977 from the CKB, and 800 from the NHS, both measuring 2,916 proteins.
Data exclusions	Among 54,219 participants with OLINK measures, we excluded 7546 participants who were manually selected with enrichment for diseases of interest, leaving 46,673 randomly selected participants. We further excluded participants with >50% missing proteins and seven proteins that were missing in over 20% of participants (GLIPR1, NPM1, PCOLCE, CST1, CTSS, TACSTD2 and ENDOU). The final discovery dataset included 43,616 participants with 2,916 proteins from the UKB. The validation datasets included 3,977 participants with 2,916 proteins from the CKB, and 800 participants with 2,916 proteins from the NHS.
Replication	We leveraged the largest proteomic dataset to date from the UK Biobank (UKB; n = 43,616) to construct proteomic aging clocks at both the organismal and organ-specific levels across 10 major organ systems, using nonlinear machine learning methods. We externally validated these models in two cohorts with distinct ethnic and geographic backgrounds: the China Kadoorie Biobank (CKB; n = 3,977) and the US-based Nurses' Health Study (NHS; n = 800).
Randomization	No randomization was required as all samples were included in the analysis.
Blinding	No blinding was applicable to this observational study as no intervention were applied to participants.

Reporting for specific materials, systems and methods

We require information from authors about some types of materials, experimental systems and methods used in many studies. Here, indicate whether each material, system or method listed is relevant to your study. If you are not sure if a list item applies to your research, read the appropriate section before selecting a response.

Materials & experimental systems

n/a	Involved in the study	
☒	☐	Antibodies
☒	☐	Eukaryotic cell lines
☒	☐	Palaeontology and archaeology
☒	☐	Animals and other organisms
☒	☐	Clinical data
☒	☐	Dual use research of concern
☒	☐	Plants

Methods

n/a	Involved in the study	
☒	☐	ChIP-seq
☒	☐	Flow cytometry
☐	☒	MRI-based neuroimaging

Plants

Seed stocks	NA
Novel plant genotypes	NA
Authentication	NA

Magnetic resonance imaging

Experimental design

Design type	Structural MRI and Diffusion MRI
Design specifications	UK Biobank brain imaging data including 6 modalities, including structural, diffusion, and functional imaging: T1-weighted structural image, resting-state functional MRI, task functional MRI, T2-weighted FLAIR structural image, diffusion MRI and susceptibility-weighted imaging.
Behavioral performance measures	NA (participants are not asked to perform tasks or behaviors during scans)

Acquisition

Imaging type(s)	T1-weighted structural imaging.
Field strength	3.0 T
Sequence & imaging parameters	The EPI-based acquisitions (dMRI, rfMRI and tfMRI) utilize simultaneous multi-slice (multiband) acceleration [Larkman et al., 2001, Moeller et al., 2010]. Biobank uses pulse sequences and reconstruction code from the Center for Magnetic Resonance Research (CMRR), University of Minnesota https://www.cmrr.umn.edu/multiband. These developments were partially generated as part of the Human Connectome Project (HCP, NIH grant 1U54MH091657), as described in [Ugurbil et al., 2013].<br><br>Resolution: 1x1x1 mm<br>Field-of-view: 208x256x256 matrix<br>Duration: 5 minutes<br>3D MPRAGE, sagittal, in-plane acceleration iPAT=2, prescan-normalise.<br><br>Full list of imaging parameters are detailed on the UK Biobank website (https://biobank.ctsu.ox.ac.uk/showcase/showcase/docs/brain_mri.pdf) and previous publications (Miller et al., Nature Neuroscience 2016)
Area of acquisition	Whole brain

Diffusion MRI ☒ Used ☐ Not used

Parameters	Resolution: 2x2x2 mm<br>Field-of-view: 104x104x72 matrix<br>Duration: 7 minutes (including 36 seconds phase-encoding reversed data)<br>5x b=0 (+3x b=0 blip-reversed), 50x b=1000 s/mm2, 50x b=2000 s/mm2<br>Gradient timings: δ=21.4 ms, Δ=45.5 ms; Spoiler b-value = 3.3 s/mm2<br>SE-EPI with x3 multislice acceleration, no iPAT, fat saturation

Preprocessing

Preprocessing software	Tissue-type and grey matter segmentation of MR images is applied using FAST (FMRIB's Automated Segmentation Tool), and subcortical structures are modelled using FIRST (FMRIB's Integrated Registration and Segmentation Tool). Grey matter volumes (GMV) of 139 cortical, subcortical, and cerebellar regions based on Harvard-Oxford atlas and Diedrichsen cerebellar atlas were then derived from T1-weighted MRI. Total white matter hyperintensity (tWMH) and microstructural measures of white matter tracts (fractional anisotropy [FA], mean diffusivity [MD], intracellular volume fraction, orientation dispersion, isotropic volume fraction) were derived from diffusion MRI.
Normalization	Tissue-type segmentation data are then used to carry out a SIENAX-style analysis (Structural Image Evaluation, using Normalisation, of Atrophy: Cross-sectional [Smith et al., 2002]). The external surface of the skull is estimated from the T1, and used to normalise brain tissue volumes for head size (compared with the MNI152 template). Volumes of different tissue types and total brain volume, both normalised for head size, and not normalised, are generated as IDPs and accessible from the UK Biobank database.
Normalization template	MNI152 "nonlinear 6th generation" standard-space T1 template http://www.bic.mni.mcgill.ca/ServicesAtlases/ICBM152NLin6.
Noise and artifact removal	Please see full details in Miller et al., Nature Neuroscience 2016.
Volume censoring	NA

Statistical modeling & inference

Model type and settings	Regression/correlation (see Methods for full details)
Effect(s) tested	Association of baseline proteomic aging gap across organ systems with GMV, tWMH, and microstructural measures of white matter tracts measured during the imgaing follow-up study (see Methods for full details)

Specify type of analysis: ☐ Whole brain ☐ ROI-based ☒ Both

Anatomical location(s): ROI (IDPs-imaging-derived phenotypes) cover the entire brain; Some visualizations of the effects voxel-wise were created.

Statistic type for inference (See Eklund et al. 2016): voxel-wise association, voxel-wise FDR association

Correction: FDR

Models & analysis

n/a	Involved in the study
☒	☐ Functional and/or effective connectivity
☒	☐ Graph analysis
☒	☐ Multivariate modeling or predictive analysis
